# Hepatitis B virus exploits C‐type lectin receptors to hijack cDC1s, cDC2s and pDCs

**DOI:** 10.1002/cti2.1208

**Published:** 2020-12-08

**Authors:** Laurissa Ouaguia, Tania Dufeu‐Duchesne, Vincent Leroy, Thomas Decaens, Jean‐Baptiste Reiser, Eleonora Sosa Cuevas, David Durantel, Jenny Valladeau‐Guilemond, Nathalie Bendriss‐Vermare, Laurence Chaperot, Caroline Aspord

**Affiliations:** ^1^ Institute for Advanced Biosciences, Immunobiology and Immunotherapy in Chronic Diseases Inserm U 1209 CNRS UMR 5309 Université Grenoble Alpes Grenoble France; ^2^ R&D Laboratory Etablissement Français du Sang Auvergne‐Rhône‐Alpes Grenoble France; ^3^ Hepato‐Gastroenterology Unit CHU Grenoble Alpes Grenoble France; ^4^ Institute for Advanced Biosciences Research Center Inserm U1209/CNRS 5309/UGA Analytic Immunology of Chronic Pathologies La Tronche France; ^5^ Université Grenoble Alpes Grenoble France; ^6^ Institut de Biologie Structurale CNRS CEA Université Grenoble Alpes Grenoble France; ^7^ INSERM 1052 CNRS 5286 Centre Léon Bérard Centre de Recherche en Cancérologie de Lyon Université Lyon Université Claude Bernard Lyon 1 Lyon France

**Keywords:** C‐type lectin receptor, DC subsets, glycans, HBsAg, hepatitis B virus, immune subversion

## Abstract

**Objectives:**

C‐type lectin receptors (CLRs) are key receptors used by DCs to orchestrate responses to pathogens. During infections, the glycan–lectin interactions shape the virus–host interplay and viruses can subvert the function of CLRs to escape antiviral immunity. Recognition of virus/viral components and uptake by CLRs together with subsequent signalling cascades are crucial in initiating and shaping antiviral immunity, and decisive in the outcome of infection. Yet, the interaction of hepatitis B virus (HBV) with CLRs remains largely unknown. As HBV hijacks DC subsets and viral antigens harbour glycan motifs, we hypothesised that HBV may subvert DCs through CLR binding.

**Methods:**

We investigated here the pattern of CLR expression on BDCA1^+^ cDC2s, BDCA2^+^ pDCs and BDCA3^+^ cDC1s from both blood and liver of HBV‐infected patients and explored the ability of HBsAg to bind DC subsets through specific CLRs.

**Results:**

We highlighted for the first time that the CLR repertoire of circulating and intrahepatic cDC2s, cDC1s and pDCs was perturbed in patients with chronic HBV infection and that some CLR expression levels correlated with plasma HBsAg and HBV DNA levels. We also identified candidate CLR responsible for HBsAg binding to cDCs (CD367/DCIR/CLEC4A, CD32/FcɣRIIA) and pDCs (CD369/DECTIN1/CLEC7A, CD336/NKp44) and demonstrated that HBsAg inhibited DC functions in a CLR‐ and glycosylation‐dependent manner.

**Conclusion:**

HBV may exploit CLR pathways to hijack DC subsets and escape from immune control. Such advances bring insights into the mechanisms by which HBV subverts immunity and pave the way for developing innovative therapeutic strategies to restore an efficient immune control of the infection by manipulating the viral glycan–lectin axis.

## Graphical abstract

Dendritic cells (DCs) are crucial in orchestrating immunity to pathogens. Sensing of virus/viral components and uptake by C‐type lectin receptors (CLRs) expressed by DCs initiate and shape antiviral immunity and are decisive in the outcome of infection. We demonstrate that hepatitis B virus exploits CLR pathways to hijack DC subsets and escape from immune control, paving the way for developing innovative antiviral approaches by manipulating the viral glycan–lectin axis.

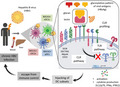

## Introduction

Dendritic cells (DCs) are professional antigen‐presenting cells (APCs) specialised in the orchestration of antiviral immune responses.^1^, ^2^ DCs own a unique ability to detect invading pathogens, capture and cross‐present viral antigens to effector cells and provide additional signals that trigger innate and adaptive immune responses against viral infections. DCs exploit different sensors that recognise pathogen‐associated molecular patterns (PAMPs) through specific pattern recognition receptors (PRRs) such as Toll‐like receptors (TLRs), nucleotide‐binding oligomerisation domains‐like receptors (NLRs), retinoic acid‐inducible gene‐I like receptors (RLRs), cytosolic DNA sensors (CDSs) and C‐type lectin receptors (CLRs).^3^ Following PAMP recognition, DCs integrate all signalling pathways and translate them into adaptive immune responses through production of antiviral cytokines especially type I and III interferons (IFNs) together with IL12p70 and cooperation with immune effectors, thereby shaping the outcome of antiviral responses. There are specialised DC subsets that differ in ontology, localisation, surface marker expression, molecular phenotype, cytokine production, and antigen‐processing and presentation capacity.^4^ Three major DC subsets exist in human blood and are also found in liver^5^, ^6^, ^7^: myeloid or conventional CD11c^+^ DCs (cDCs) subdivided into two subsets based on the differential expression of CD1c/BDCA1 (cDC2s) and CD141/BDCA3 (cDC1s) molecules,^4^ and plasmacytoid DCs (pDCs) which are CD11c^–^ BDCA2^+^ BDCA4^+^ ^8^. Each DC subset displays its own repertoire of TLRs and a specific pattern of CLRs that endowed them with a functional specialisation and tightly regulate their response.^9^, ^10^

C‐type lectin receptors are part of the PRRs specialised in the sensing and binding of carbohydrate structures (glycans) present within glycolipids and glycoproteins on the surface of a variety of pathogens, including viruses.^11^ This recognition process is based on the presence of a carbohydrate recognition domain in the C‐terminus of CLR that sense specific carbohydrate structures in a Ca^2^^+^‐dependent manner.^12^, ^13^ CLR engagement by viral glycans on DCs leads to virus internalisation and cross‐priming of T‐cell responses, but also triggers signalling cascades leading to expression of co‐stimulatory molecules and production of cytokines, contributing to the shaping and polarisation of antiviral immune responses.^9^, ^14^, ^15^ Depending on the signalling motifs in their cytoplasmic portion, CLRs are classified into four main groups^13^, ^14^, ^15^: immunoreceptor tyrosine‐based activation motif (ITAM)‐coupled CLRs (DECTIN2/CLEC6A), hemi‐ITAM (hemITAM)‐bearing CLRs (DECTIN1/CD369/CLEC7A, DNGR1/CD370/CLEC9A) (both trigger activation kinases such as SYK, recruitment of CARD9 leading to activation of transcription factors such as NFкB ultimately leading to cytokine production and T‐cell priming), immunoreceptor tyrosine‐based inhibitory motif (ITIM)‐containing CLRs (DCIR/CD367/CLEC4A, CD371/CLEC12A) (inhibition of cellular responses through recruitment of tyrosine phosphatases SHP‐1/2), and CLRs lacking typical signalling motifs (MMR/CD206, DEC205/CD205, DC‐SIGN/CD209, langerin/CD207, BDCA2/CD303/CLEC4C) (involved in endocytosis and contributing to antigen processing and presentation to T cells) but that can recruit an adaptor protein that contains ITAM such as FcγRIIA/CD32/.^13^, ^14^ BDCA2/CD303/CLEC4C,^16^ ILT7/CD85g,^17^ and NKp44/CD336^18^ are uniquely expressed on human pDCs and paradoxically potently suppress their ability to produce type I IFN in response to TLR7/9 triggering despite signalling through ITAMs due to the use of BCR‐like signalling cascades.

C‐type lectin receptors ligands are often complex structures that simultaneously bind different CLRs and other PRRs.^15^ Moreover, specific features of the ligand (affinity, avidity) may result in distinct signallings through a single motif. Multimerisation of CLRs forming homo‐ or hetero‐complexes facilitates a cooperative response to the ligand. Several CLRs (DC‐SIGN, DCIR, BDCA2) can also affect signalling triggered by other PRRs, especially TLRs. Thus CLR signalling pathways are highly flexible and can trigger diverse responses depending on the carbohydrate‐specific pathways, cross‐talk with other PRRs, and the cell type (including DC subsets) that integrates all these signals. During viral infections, the glycan–lectin interactions shape virus–host interplay, and are crucial in triggering subsequent antiviral immunity and determining the outcome of infection.^15^ Indeed, upon recognition of viruses, CLRs can elicit adaptive immunity,^19^ yet certain viruses exploit CLRs for entry into host cells to avoid immune recognition, promote their spreading, disrupt signalling pathways, inhibit APC functions or drive immune suppression.^12^, ^13^, ^20^ Several interactions between viral proteins and CLRs have already been described. DC‐SIGN and DCIR can mediate HIV1 capture and transfer by DCs by interacting with gp120.^21^, ^22^, ^23^ HCV envelope glycoprotein E2 can bind to BDCA2 and DCIR, inhibiting IFNα production by pDCs.^24^ Clec5a interacts with the dengue virion, stimulating the release of pro‐inflammatory cytokines and subsequent lethal disease.^25^ Modifications of glycan structures on pathogens/viruses to mimic host glycans can alter CLR interactions that subsequently modify DC‐induced polarisation and immune response.^11^ Therefore, CLRs have a dual function, being crucial in viral clearance but also being exploited by pathogens to escape immunity.

Hepatitis B virus (HBV) is a double‐stranded DNA virus, which specifically infects hepatocytes and that can cause chronic liver diseases such as cirrhosis, liver failure and hepatocarcinoma^26^ being therefore a major health burden.^27^ The natural history of HBV infection is the result of complex interactions between the replicating non‐cytopathic virus and the host immune system.^28^ Infected hepatocytes produce infectious viral particles as well as large amount of other ‘particle entities’, including Sub‐Viral Particles (SVPs) that are composed by a host bilayer of lipids in which envelope proteins (mainly M and S, but some L protein is also found) are densely incorporated, but do not contain the viral nucleocapsid. SVPs out‐number infectious particles by a factor of 10 to 10 000 fold according to the phase of the disease.^26^, ^29^ Collectively, infectious particles (also called Dane particles), RNA‐positive particles, genome free‐particles, and SVPs define the circulating HBs antigen (HBsAg), which can be clinically dosed to monitor ongoing infection. Whereas patients who clear the infection elicit broad and potent humoral responses and cytotoxic effectors able to eliminate the virus and infected cells, patients who evolve towards chronic infection display weak and inappropriate responses. The physiopathology of HBV is strongly related to the host immunity, yet the mechanisms of HBV sensing by APCs and subsequent modulation of the immune system are still poorly understood. Despite the crucial role of DCs in orientating antiviral responses and determining the outcome of infection, their precise involvement in HBV pathogenesis is not fully understood. We and others demonstrated major functional alterations of circulating and intrahepatic DC subsets (BDCA1^+^ cDC2s, BDCA2^+^ pDCs, and BDCA3^+^ cDC1s) in the context of chronic HBV infection.^30^, ^31^, ^32^, ^33^, ^34^, ^35^, ^36^ HBV virions and viral antigens have been detected within cDC2s^37^ and pDCs^38^ from chronic HBV patients. Most studies demonstrate that HBV does not trigger a direct activation of DCs but impairs their functionality.^30^, ^31^, ^34^ Circulating cDC2s from HBV patients display impairment in their maturation associated with a defective IL‐12 production upon stimulation.^32^, ^37^ Viral particles or HBs/HBc viral antigens have also been found within pDCs from chronic HBV patients,^38^ suggesting direct interactions between HBV and pDCs. We previously reported *ex vivo* modulations of the activation status of circulating and intrahepatic pDCs from chronic HBV patients, associated with an altered OX40L expression and IFNα production in response to TLR9 triggering leading to a defective triggering of NK cytotoxic effectors.^35^ Alterations of pDC functions in HBV patients could be linked to the binding of HBsAg to BDCA2^34^ or to the impairment of TLR9 expression.^39^ Regarding cDC1s which are prominently present in HBV‐infected liver, Woltman *et al*. reported an impaired maturation together with reduced IFNλ1 production by blood cDC1s from chronic HBV patients after TLR3 triggering.^31^ Thus, HBV triggers dysfunctional immune responses, but the mechanism of escape from immune control remains unclear. The early steps in the recognition of HBV by immune cells, especially the receptors involved in the binding of HBV and/or HBV antigens on DCs, and the functional consequences of such interaction remain unknown.

Recognition and uptake of virus by CLRs together with subsequent signalling cascades are decisive in the outcome of infection, yet the interaction of HBV with CLRs is largely unknown. As HBV hijacks DC subsets,^30^ that is, exploits DCs' plasticity and competencies to its own advantage and harbours glycan patterns,^40^ we hypothesise that HBV particles may subvert DCs through CLR binding. We investigated here the pattern of CLRs expression on BDCA1^+^ cDC2s, BDCA3^+^ cDC1s, and BDCA2^+^ pDCs from both blood and liver of HBV‐infected patients and assessed their clinical relevance. We further explored the ability of HBsAg to bind each DC subset and determined the CLRs involved in such attachment. We finally evaluated the impact of CLR‐dependent binding of HBsAg on the function of each DC subset. Our findings highlight for the first time that HBV/HBV‐derived antigens trigger major perturbation of CLR profiles on circulating and intrahepatic DC subsets, and we identify candidate CLRs responsible for HBsAg binding to DCs. HBV may exploit CLR pathways to hijack DC subsets and escape from immune control. These results bring insight into the mechanisms by which HBV subverts immunity and pave the way for manipulating the viral glycan–lectin axis to restore an efficient immune control of the infection.

## Results

### Peripheral and intrahepatic BDCA1^+^ cDC2, BDCA3^+^ cDC1, and BDCA4^+^ pDCs from chronic HBV patients display modulation of their basal CLR expression

As DCs are crucial in binding carbohydrate moieties of pathogens to mediate immune responses, we investigated the basal expression of specific CLR molecules among peripheral and intrahepatic cDCs and pDCs. We designed a multiparametric flow cytometry strategy allowing the simultaneous and extensive analysis of specific CLR molecules of the three major DC subsets, from blood and liver samples of HBV patients and healthy donors (HD) or non‐viral infected controls (Supplementary figure 1). Among CD45^+^ cells within fresh Peripheral blood mononuclear cells (PBMCs) or liver mononuclear cells (LMNCs), BDCA1^+^ cDC2s were defined to be Lin^neg^HLA‐DR^+^CD11c^+^ BDCA1^+^ cells, BDCA3^high^Clec9A^+^ cDC1s picked out as Lin^neg^HLA‐DR^+^CD11c^+^ BDCA3^high^Clec9A^+^ cells, and pDCs identified as Lin^neg^HLA‐DR^+^CD11c^neg^BDCA1^neg^BDCA4^+^ cells (Supplementary figure 1a). Once gated on each specific DC subset, we then analysed the expression of specific membrane‐associated CLRs (Supplementary figure 1b).

**Figure 1:**
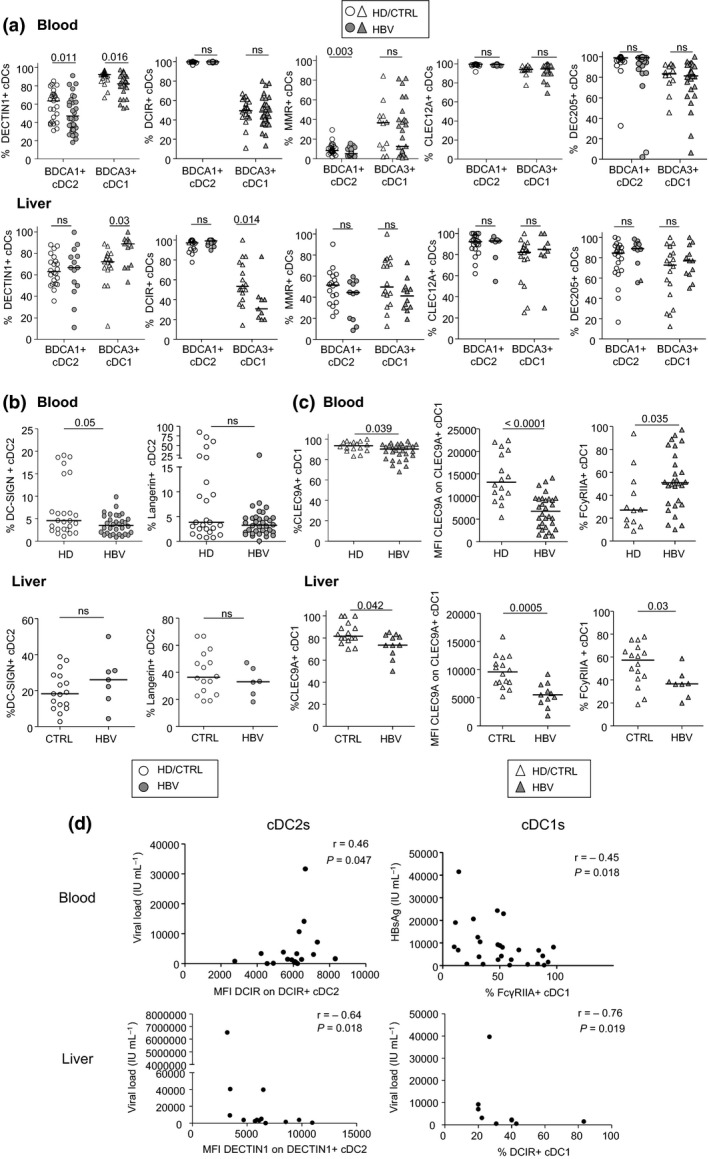
Circulating and intrahepatic cDC2s and cDC1s from chronic HBV patients display perturbation of their basal CLR expression. The expression of CLR molecules by cDC2s and cDC1s was analysed by flow cytometry from fresh PBMCs and LMNCs derived from chronic HBV patients and HD or non‐viral infected controls. DC subsets were identified using specific markers (see Supplementary figure 1). **(a)** Expression levels of shared CLR on circulating (upper panels) and intrahepatic (lower panels) cDC2s and cDC1s: DECTIN1, DCIR, MMR, CLEC12A and DEC205. **(b)** Expression levels of DC‐SIGN and Langerin on circulating (upper panels) and intrahepatic (lower panels) cDC2s. **(c)** Expression levels of CLEC9A and FcɣRIIA on circulating (upper panels) and intrahepatic (lower panels) cDC1s. Results are expressed as percentages or MFI of positive cells within the corresponding subset. Bars indicate median. Blood: open symbols, HD (*n* = 12–25) and filled symbols, HBV patients (*n* = 29–34); liver: open symbols, non‐infected controls (*n* = 17–22) and filled symbols, HBV patients (*n* = 7–14). *P*‐values were calculated using the Mann–Whitney test. **(d)** Upper panels: Spearman’s correlation of DCIR expression (MFI) on circulating cDC2s with HBV DNA levels (*n* = 17) and FcɣRIIA expression (%) on circulating cDC1s with HBsAg levels (*n* = 28); lower panels: Spearman's correlations of DECTIN1 expression (MFI) and DCIR expression (%) on intrahepatic cDC2s (*n* = 13) or cDC1s (*n* = 8) respectively with HBV DNA levels.

**Figure 2:**
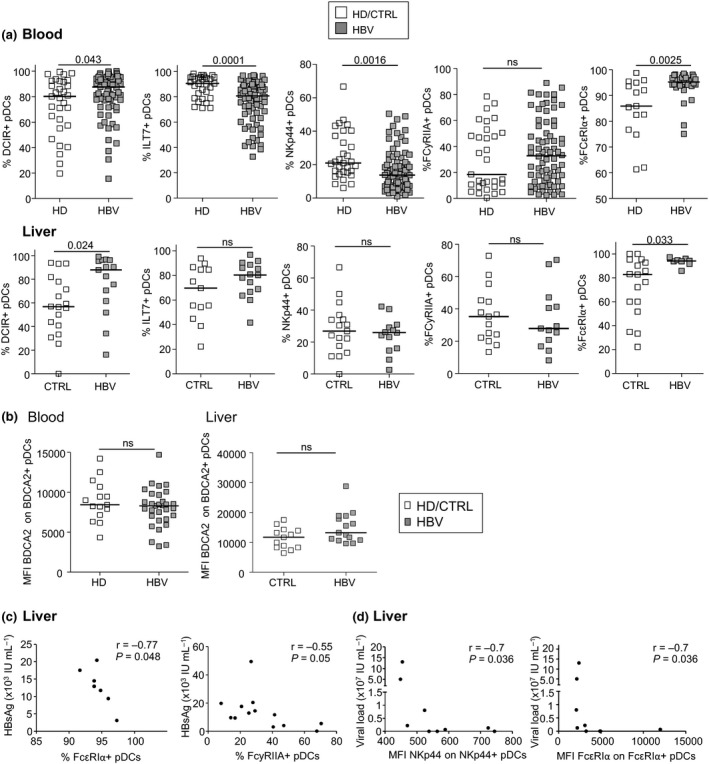
Circulating and intrahepatic pDCs from chronic HBV patients display modulation of their basal CLR expression. The expression of CLR molecules by pDCs was analysed by flow cytometry from fresh PBMCs and LMNCs derived from chronic HBV patients and HD or non‐viral infected controls. pDCs were identified using specific markers (see Supplementary figure 1). **(a)** Expression levels of DCIR, ILT7, NKp44, FcɣRIIA and FcεRIα by circulating (upper panels) and intrahepatic (lower panels) pDCs. Results are expressed as percentages of positive cells within pDCs. Bars indicate median. Blood: open symbols, HD (*n* = 15–32) and filled symbols, HBV patients (*n* = 31–72); liver: open symbols, controls (*n* = 13–18) and filled symbols, HBV patients (*n* = 8–15). *P*‐values were calculated using the Mann–Whitney test. **(b)** Expression levels of BDCA2 on circulating and intrahepatic pDCs. Results are expressed as MFI of BDCA2 on positive cells. Bars indicate median. Blood: open symbols, HD (*n* = 15) and filled symbols, HBV patients (*n* = 32). Liver: open symbols, controls (*n* = 13) and filled symbols, HBV patients (*n* = 15). *P*‐values were calculated using the Mann–Whitney test. **(c)** Spearman's correlations of FcεRIα and FcɣRIIA expression (%) on intrahepatic pDCs with HBsAg levels in chronic HBV patients (*n* = 7–13). **(d)** Spearman's correlations of NKp44 and FcεRIα expression (MFI) on intrahepatic pDCs with viral load levels from chronic HBV patients (*n* = 8 or 9).

Evaluation of the proportion of cDC2s, cDC1s, and pDCs revealed no difference in the circulating DC frequencies between HBV patients and controls (Supplementary figure 2a). However, our results showed a significant increased frequency of intrahepatic pDC proportion in HBV context, while similar proportions of liver cDCs were observed (Supplementary figure 2a), suggesting a specific accumulation of pDCs in the liver of chronically infected HBV patients. The investigation of the basal expression of specific CLRs and adaptors on cDCs (Figure 1, Supplementary figures 2b and c and 3) and pDCs (Figure 2 and Supplementary figure 4) revealed perturbations of CLR expression by circulating and intrahepatic DC subsets in HBV context. Circulating cDC2s from HBV patients displayed reduced expression of DECTIN1 and MMR compared with HD, while intrahepatic cDC2s exhibited a reduced expression of DCIR (MFI) and MMR (MFI) compared to controls (Figure 1a and Supplementary figure 2c). Circulating cDC1s from HBV patients displayed reduced expressions of DECTIN1 (Figure 1a), CLEC9A (Figure 1c) together with increased expression of FcɣRIIA (Figure 1c and Supplementary figure 2b) compared with HD, while intrahepatic cDC1s exhibited a reduced expression of DCIR (Figure 1a), CLEC9A, FcɣRIIA, (Figure 1c) and MMR (MFI) (Supplementary figure 2c) together with increased expression of DECTIN1 (Figure 1a) compared to controls. Interestingly, modulations of CLR patterns on pDCs are more homogeneous than on cDCs, with downregulation and upregulation of CLR expression in the circulation and liver respectively. Importantly, we observed a significant decrease of ILT7 and NKp44 expression on circulating pDCs from HBV patients compared to HD, while both circulating and intrahepatic pDCs displayed higher expression of DCIR and FcεRIα compared to controls (Figure 2a and Supplementary figure 3a and b). There was no significant modulation of BDCA2 expression between the groups (Figure 2b). Taken together, these results indicate for the first time that specific CLR molecules are modulated on circulating and intrahepatic DC subsets in chronic HBV patients. cDC2s are mostly impacted in the circulation, while cDC1s mostly hampered in the liver, and pDCs modulated both in blood and liver.

### Relations between the skewed CLR profile and viral parameters in HBV patients

In order to decipher the clinical relevance of these observations, we performed correlations between CLR expression on specific DC subsets and clinical and viral parameters of the corresponding patients for both circulating cDCs and pDCs (Supplementary table 2, Figure 1d and Supplementary figure 4) and intrahepatic cDCs and pDCs (Supplementary table 3, Figures 1d, 2c and d, Supplementary figures 3c and 4). We observed that the CLR expression profile among blood and liver DCs from HBV patients correlated with clinical viral parameters (ALT, Viral load, and HBsAg levels). Negative correlations were observed in blood between proportions of DECTIN1 or FcɣRIIA**‐**positive circulating DCs and HBsAg/HBV DNA levels, while positive correlations occurred between proportions of DEC205 and viral related parameters. When considering CLRs disrupted in HBV patients compared to HD, their expression on circulating DC subsets according to plasmatic HBsAg levels revealed that DECTIN1 and/or FcɣRIIA tends to decrease on cDC2s and cDC1s (Supplementary figure 5a and b), whereas ILT7 and NKp44 tends to increase on pDCs (Supplementary figure 5c) in patients with high HBsAg level. These observations support that DCs may sense HBsAg level through DECTIN1, FcɣRIIA, ILT7 or NKp44, creating a dynamic of CLR internalisation/recycling process. However, in liver, we observed exclusively strong negative correlations between the expression levels of DCIR, DECTIN1, MMR, NKp44, FcɣRIIA, and FcεRIα on DC subsets and HBsAg/HBV DNA levels. As older people with HBV are more likely to have lower HBsAg levels and alterations in immune cell function compared to younger individuals, we analysed in our cohorts the impact of age on HBsAg level and on CLR expression (Supplementary figure 6). Whereas HBsAg levels were similar between old and young patients for the liver cohort, we actually observed significant lower HBsAg levels in older patients for the blood cohort (Supplementary figure 6a). However, such impact did not affect the expression level of the CLRs for which we observed a clinical correlation, as levels of DECTIN1, DEC205, FcɣRIIA, and MMR on circulating DCs were not significantly impacted by the age of the patients (Supplementary figure 6b). Therefore, the observed associations between HBsAg levels and CLR profile are independent of the age of the patients. Thus, HBV or HBV**‐**derived antigens alter the CLR expression profile and dynamic on circulating and intrahepatic cDC2s, cDC1s, and pDCs, suggesting potential interactions between HBV and the CLR machinery of DCs.

### HBsAg viral protein strongly impacts CLR expression on pDCs from healthy controls, without any major effects on cDCs in an acute setting

**Figure 3:**
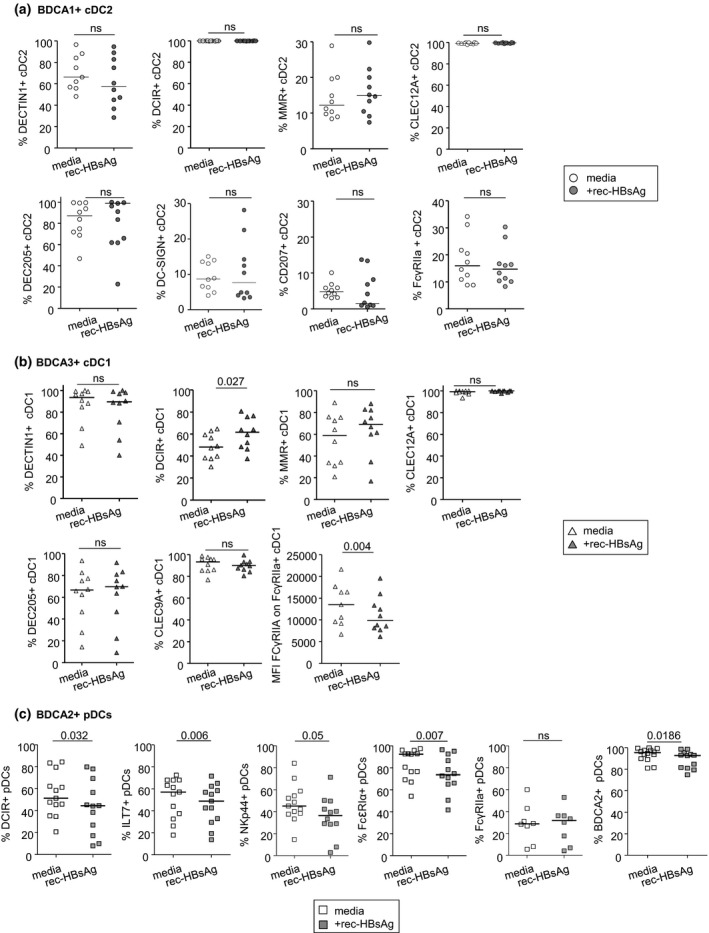
HBsAg modulates CLR expression on DC subsets. Purified pDCs or total PBMCs were cultured for 4 h with or without rec‐HBsAg (25 μg mL^−1^). The modulation of CLR expression on each DC subset was measured by flow cytometry. **(a)** Comparative expression of DECTIN1, DCIR, MMR, CLEC12A, DEC205, DC‐SIGN, Langerin and FcɣRIIA on cDC2s. **(b)** Comparative expression of DECTIN1, DCIR, MMR, CLEC12A, DEC205, CLEC9A and FcɣRIIA on cDC1s. **(c)** Comparative expression of DCIR, ILT7, NKp44, FcεRIα, FcɣRIIA and BDCA2 on pDCs. Results are expressed as % or MFI of positive cells. Bars indicate median. Open symbols, media (*n* = 8–13); filled symbols, +rec‐HBsAg (*n* = 8–10). *P*‐values were calculated using the Wilcoxon paired *t*‐test. *N* = 3 independent experiments.

The previous results prompted us to investigate whether HBsAg could directly affect CLR expression on DC subsets. We therefore studied *ex vivo* the impact of the recombinant HBsAg protein (rec**‐**HBsAg) on the above studied CLR expression on BDCA1^+^ cDC2s, BDCA3^high^CLEC9A^+^ cDC1s, and BDCA4^+^ pDCs within PBMCs from HDs (Figure 3 and Supplementary figure 7). Contrary to the previous analysis on patients’ DCs that reflected CLR patterns upon a chronic exposure, we assessed the CLR profile on healthy DCs after short term 4‐h incubation. The dose of 25 µg mL^−1^ of HBsAg corresponds to about 10 000 UI mL^−1^, a level commonly observed in the circulation of HBV patients, either viraemic or treated with nucleos(t)ide analogs (NUC). In these ‘acute’ settings, rec**‐**HBsAg did not impact CLR expression on cDC2s, while it triggered a significant increase in DCIR expression together with a decrease in FcɣRIIA expression on cDC1s (Figure 3a andb and Supplementary figure 7a). Notably, rec**‐**HBsAg drove major modulations of CLR expression on pDCs, which is consistent with observations in patients where the CLR expression pattern was much more perturbed in pDCs compared to cDCs. We depicted here a significant downregulation of DCIR, ILT7, NKp44, FcεRIα, and BDCA2 molecule expression on pDCs compared to control conditions (Figure 3c and Supplementary figure 7b). We then further assessed on pDCs whether such CLR modulations were dose‐dependent and still occurred upon activation of pDCs with TLR‐L. We observed that the impact of rec**‐**HBsAg on CLR expression on pDCs was concentration‐dependent but was abrogated upon TLR‐L stimulation (Supplementary figure 7c). Taken together, these results demonstrated that HBsAg viral protein is able to specifically alter CLR expression on DC subsets in an ‘acute’ setting, suggesting that HBV may interact with DCs through CLR molecules.

### The viral protein HBsAg binds to cDC2s, cDC1s, and pDCs in a glycan‐dependent manner

**Figure 4:**
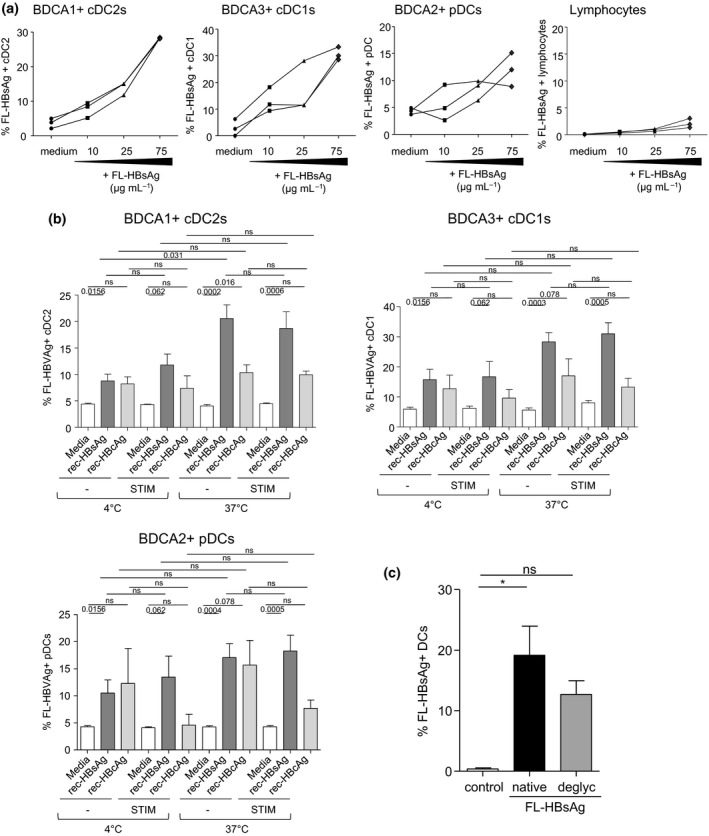
HBsAg binds on cDC2, cDC1 and pDCs in a dose‐ and glycan‐dependent manner. Rec‐HBsAg and rec‐HBcAg were labelled with DyLight 650 sulfhydryl‐reactive dye. PBMCs were cultured for 2 h at 4 or 37°C in presence or not of FL‐HBsAg or FL‐HBcAg at various concentrations and with or without a mixture of TLRL (stim: polyI:C+R848+CpG_A_). The binding/fixation of FL‐HBV‐Ag on DC subsets was then measured by flow cytometry. **(a)** FL‐HBsAg‐positive cDC2s, cDC1s, pDCs and lymphocytes upon incubation of PBMCs with 10, 25 or 75 μg mL^−1^ of FL‐HBsAg. Results are expressed as % of FL‐HBsAg‐positive cells (*n* = 1 experiment with three donors). **(b)** FL‐HBsAg and FL‐HBcAg labelling on cDC2s, cDC1s and pDCs upon incubation of PBMCs with 25 μg mL^−1^ of FL‐HBsAg or FL‐HBcAg. Results are expressed as % of FL‐HBV‐Ag‐positive cells within the corresponding DC subset. Bars indicate mean ± SEM. White bars, media (*n* = 9–14); dark grey bars, +FL‐HBsAg (*n* = 9–22); light grey bars, +FL‐HBcAg (*n* = 9–12) (pool of four independent experiments). **(c)** Comparative fixation of FL‐HBsAg on DCs upon deglycosylation (deglyc) or not (native) (*n* = 5 donors). *P*‐values were calculated using the Wilcoxon paired *t*‐test with Bonferroni correction. * *P* < 0.05

As the CLR expression pattern on both cDCs and pDCs was disrupted in patients with chronic HBV infection, we next investigated whether HBV**‐**derived antigens could bind to DC subsets. To assess such hypothesis, we synthetised fluorescence**‐**labelled**‐**recombinant**‐**HBsAg (FL‐HBsAg) and tested its ability to interact with DC subsets at 4°C and 37°C in presence or not of a mixture of TLR**‐**L (to activate each DC subset). We used fluorescence‐labelled‐recombinant‐HBcAg (FL‐HBcAg) as control, as HBcAg is known to not alter DC functions on the contrary to HBsAg. In these settings, FL‐HBs/cAg‐positive DCs will reveal the binding of viral antigens to the DCs. We highlighted for the first time the ability of FL‐HBsAg to bind specifically on BDCA1^+^ cDC2s, BDCA3^+^ cDC1s, and BDCA2^+^ pDCs within PBMCs of HD in a dose‐dependent manner (Figure 4a andb and Supplementary figure 8). FL‐HBsAg binding was improved at 37°C compared to 4°C conditions especially for cDC2s and in a lesser extend for cDC1s and pDCs, which might be explained by continuous CLR recycling allowing for accumulated antigen uptake and further argue for the specificity of HBsAg binding. Furthermore, the activation of DC subsets using a mixture of TLR‐L did not impact the binding of FL‐HBsAg on DCs (Figure 4b), suggesting that the binding of HBsAg on DCs might be independent of the activation status of the DCs. HBcAg slightly binds to DC subsets but without reaching significance (Figure 4b). Notably, the ability of FL‐HBsAg to bind to DCs was downregulated upon deglycosylation (Figure 4c), revealing that the interaction between HBsAg and DCs is dependent on the glycosylation pattern of HBsAg. Importantly, FL‐HBsAg did not affect cell viability whatever its concentration (Supplementary figure 9a). Notably, the level of HBsAg binding was lower on pDCs compared to cDCs, whereas our previous observations highlighted a stronger perturbation of CLR pattern on this subset both in acute and chronic settings, suggesting the absence of direct relationship between CLR expression level and ability to bind HBV antigens. When analysing other immune subpopulations within PBMCs for their ability to bind FL‐HBsAg, we observed no labelling of the total lymphocyte population (Figure 4a), which attested the specificity of the binding observed on DC subsets, while we noticed a significant labelling on the monocyte population (Supplementary figure 9b). Taken together, these results demonstrate for the first time that HBsAg can specifically bind all DC subsets in a glycan‐dependent manner, strengthening the fact that HBV may escape immunity by hijacking DCs.

### DCIR and FcɣRIIA versus NKp44 and DECTIN1 are candidate CLRs involved in the binding of HBsAg on cDCs and pDCs respectively

**Figure 5:**
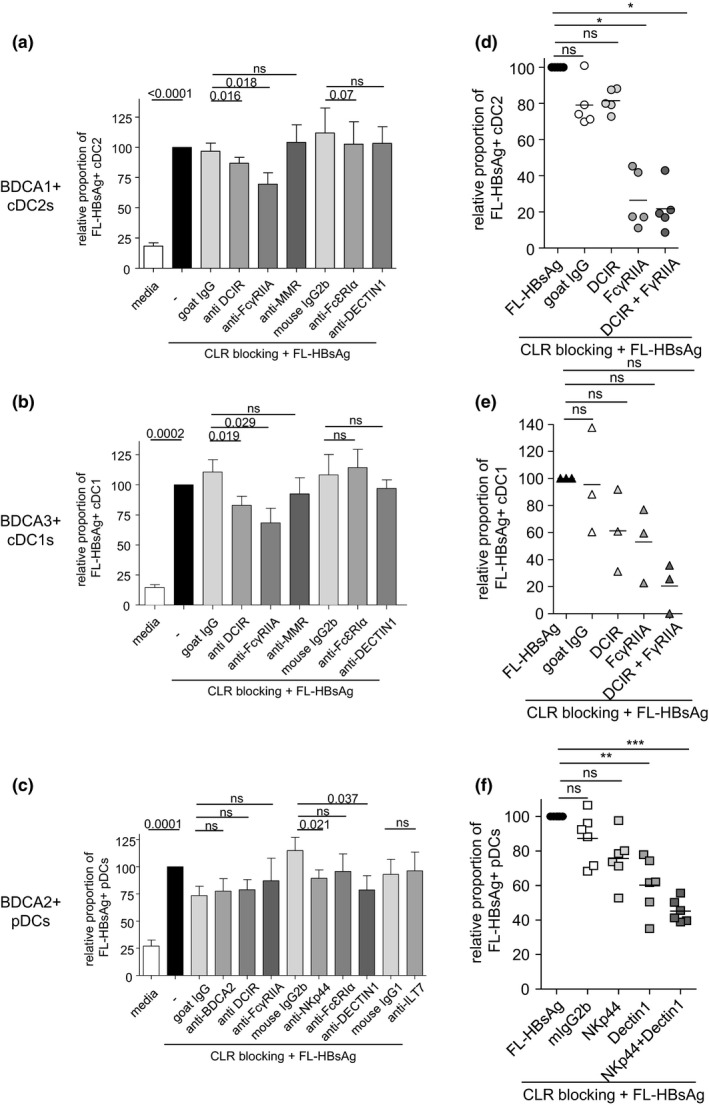
HBsAg binds cDC1s and cDC2s through DCIR and FcɣRIIA, and pDCs through DECTIN1 and NKp44. Rec‐HBsAg was labelled with DyLight 650 sulfhydryl‐reactive dye. PBMCs were cultured 30 min at 37°C with blocking anti‐DCIR, anti‐FcɣRIIA, anti‐MMR, anti‐FcεRIα, anti‐DECTIN1, anti‐NKp44, anti‐ILT7 or anti‐BDCA2 Abs, alone or in combination. FL‐HBsAg (25 μg mL^−1^) was then added or not in the solution, and the culture was incubated for 2 h at 37°C. The binding/fixation of FL‐HBsAg on DC subsets was then measured by flow cytometry. Relative proportions of FL‐HBsAg‐positive **(a)** cDC2s, **(b)** cDC1s and **(c)** pDCs, after CLR blocking. Bars indicate mean ± SEM. White bars, media; black bars, FL‐HBsAg; grey bars, anti‐CLR blocking Abs+FL‐HBsAg (*n* = 7–18). Proportions were standardised to the condition FL‐HBsAg without CLR blocking. *P*‐values were calculated using the Wilcoxon paired *t*‐test. **(d–f)** Combined blocking of several CLRs. Relative proportions of FL‐HBsAg‐positive cDC2s **(d)** and cDC1s **(e)** upon single or combined blocking of DCIR and FcγRIIA (*n* = 3–5), and of pDCs **(f)** upon single or combined blocking of Dectin1 and NKp44 (*n* = 6). *P*‐values were calculated using the ANOVA test. *P < 0.05, **P < 0.01, ***P < 0.001.

All previous observations led us to hypothesise that HBV/ HBV‐derived antigens may bind DCs through CLR. Indeed, CLRs, whose expression profile is disrupted in DCs from HBV patients, have been shown to recognise specific glycans exposed on pathogens or viruses. To determine the involvement of CLRs as putative attachment factors for HBsAg on DCs, we assessed the impact of blocking CLR on HBsAg attachment to DC subsets using specific functional grade anti‐human CLR antibodies before incubating PBMCs with FL‐HBsAg (Figure 5). Notably, the blocking of DCIR and FcɣRIIA significantly impaired the binding of FL‐HBsAg on both BDCA1^+^ cDC2s (Figure 5a) and BDCA3^+^ cDC1s (Figure 5b), while MMR, FcεRIα, and DECTIN1 were not involved. Furthermore, the blocking of NKp44 and DECTIN1 significantly affected the binding of FL‐HBsAg on pDCs (Figure 5c), while BDCA2, DCIR, FcɣRIIA, FcεRIα, and ILT7 were not involved. We further investigated whether blocking the potential CLR candidates together would further increase this effect. We observed that the combined blocking of DCIR and FcɣRIIA led to 80% of inhibition of HBsAg attachment to cDC2s and cDC1s (Figure 5d ande), whereas the simultaneous blocking of NKp44 and DECTIN1 further inhibited the binding of HBsAg to pDCs to 60% (Figure 5f). Thus, these data suggest that DCIR and FcɣRIIA are involved in the binding of HBsAg on cDCs, while NKp44 and DECTIN1 are the main CLRs involved in the binding of HBsAg on pDCs.

### HBsAg impairs IFNα2, IL12p70 and IFNλ1/IL‐29 secretions in response to TLR triggering of specific DC subsets in a CLR‐ and glycosylation‐dependent manner

**Figure 6:**
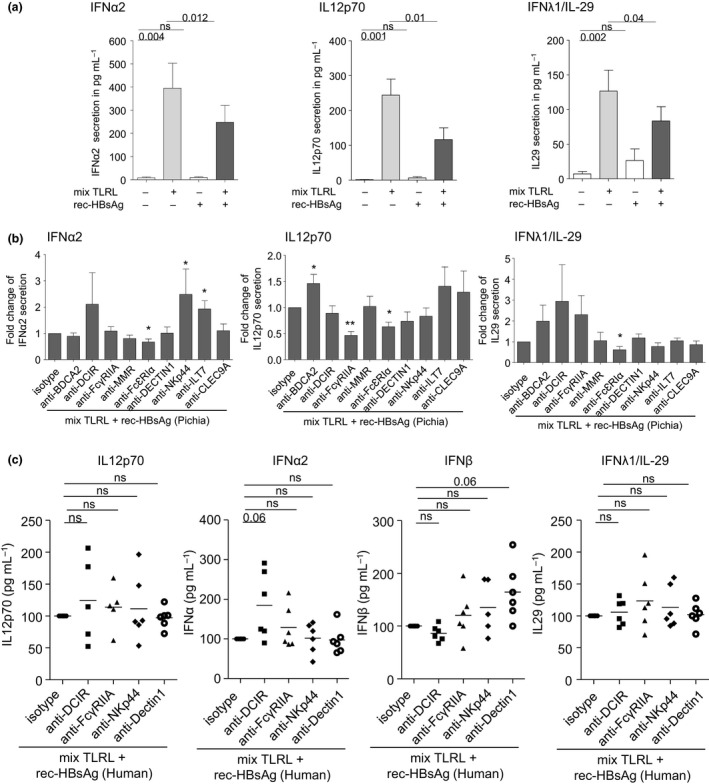
HBsAg hampers IFNα2, IL12p70 and IFNλ1/IL‐29 secretions in response to TLRL stimulation in a CLR‐dependent manner. PBMCs were cultured 30min at 37°C with or without anti‐BDCA2, anti‐DCIR, anti‐FcɣRIIA, anti‐MMR, anti‐FcεRIα, anti‐DECTIN1, anti‐NKp44, anti‐ILT7 or anti‐CLEC9A blocking Abs. Rec‐HBsAg (produced in Pichia or Human) was added or not, and cultures were incubated for 20 h in presence or not of a mixture of TLRL (MIX: polyI:C+R848+CpG_A_). Supernatants were then examined for the presence of IFNα2, IFNβ, IL‐12p70 and IFNλ1/IL‐29 by Luminex. **(a)** Evaluation of the direct impact of HBsAg (Pichia) on cytokine secretion upon TLRL stimulation (*n* = 10–11). **(b)** Assessment of the impact of CLR blocking on the modulation of cytokine secretion by HBsAg (Pichia) upon TLRL stimulation (*n* = 10–11). **(c)** Impact of specific CLR blocking (DCIR, FcγRIIA, NKp44, DECTIN1) on the modulation of cytokine secretion by HBsAg (Human) upon TLRL stimulation (*n* = 6). **(b, c)** Secretions were standardised to their corresponding isotype control condition (mIgG1, mIgG2b or goat IgG). Bars indicate mean ± SEM. *P*‐values were calculated using the Wilcoxon paired *t*‐test. *P < 0.05, **P < 0.01. For (B), only significant statistics are shown.

It has been shown that HBsAg inhibited IFNα secretion by pDCs, but its impact on the functionality of other DC subsets remained unclear or unknown. We next investigated the impact of HBsAg on the functionality of DC subsets (including cDC2s and cDC1s) together with its CLR dependency. PBMCs were cultured 30 min in presence or not of functional grade anti‐human CLR blocking antibodies before addition or not of rec‐HBsAg in presence or not of a mixture of TLRL (polyI:C+R848+CpG_A_) that allows the activation of all DC subsets. The quantification of cytokines specifically produced by DC subsets (IFNα2, IL‐12p70, and IFNλ1/IL‐29) in the different conditions allowed evaluating their functionality (Figure 6 and Supplementary figure 10). Interestingly, our data revealed that, in response to TLR triggering, rec‐HBsAg (from Pichia) strongly impaired the production of IFNα2 but also dampened the secretion of IL12p70 and IFNλ1/IL‐29 (Figure 6a). When performing these experiments upon CLR blocking, we observed that anti‐NKp44, anti‐ILT7 Abs for IFNα2, anti‐FcɣRIIA Abs for IL12p70, and anti‐FcεRIα for IFNλ1/IL‐29 significantly modulated (positively or negatively) cytokine secretion (Figure 6b) without affecting it in absence of HBsAg (Supplementary figure 10), which is in accordance with the previously observed inhibition of HBsAg binding to pDCs and cDCs upon blocking of NKp44 and FcɣRIIA respectively. This suggests that HBsAg may attach to these CLRs to modulate DC function. Importantly, such modulations of cytokine secretions in response to TLR triggering were also observed with HBsAg derived from Human after blocking of the candidate CLRs previously shown to be involved in the binding of HBsAg to DC subsets (Figure 6c). The blocking of DCIR, FcɣRIIA, NKp44 or DECTIN1 enhanced at least one DC‐specific cytokine in response to TLR triggering in presence of HBsAg, revealing that the attachment of HBsAg (harbouring human‐made glycosylation pattern) to these candidate CLRs modulated DC subsets’ function. Positive or negative impacts may result from the differential impact of CLR signalling pathways on the TLR pathway that could be inhibitor or enhancer. The other modulations observed in presence of HBsAg were also seen in absence of HBsAg, therefore being HBsAg‐independent and resulting from a modulation of the TLR‐triggered pathway by the CLR signalling pathway. The blocking of the CLRs previously identified as potential candidates to bind DC subsets do not have systematically an impact on the reversion of DC dysfunction, and some anti‐CLRs unexpectedly impacted DC subsets' function. Such discrepancies may be due to the fact that experiments relative to the functionality were performed in whole PBMCs and with a mixture of TLRL, where a DC subset, even if able to bind HBsAg, could be rescued by another DC subset within the culture or inversely affected by another DC subset independently of HBsAg binding, thus masking potential impacts of HBsAg on DC function.

**Figure 7:**
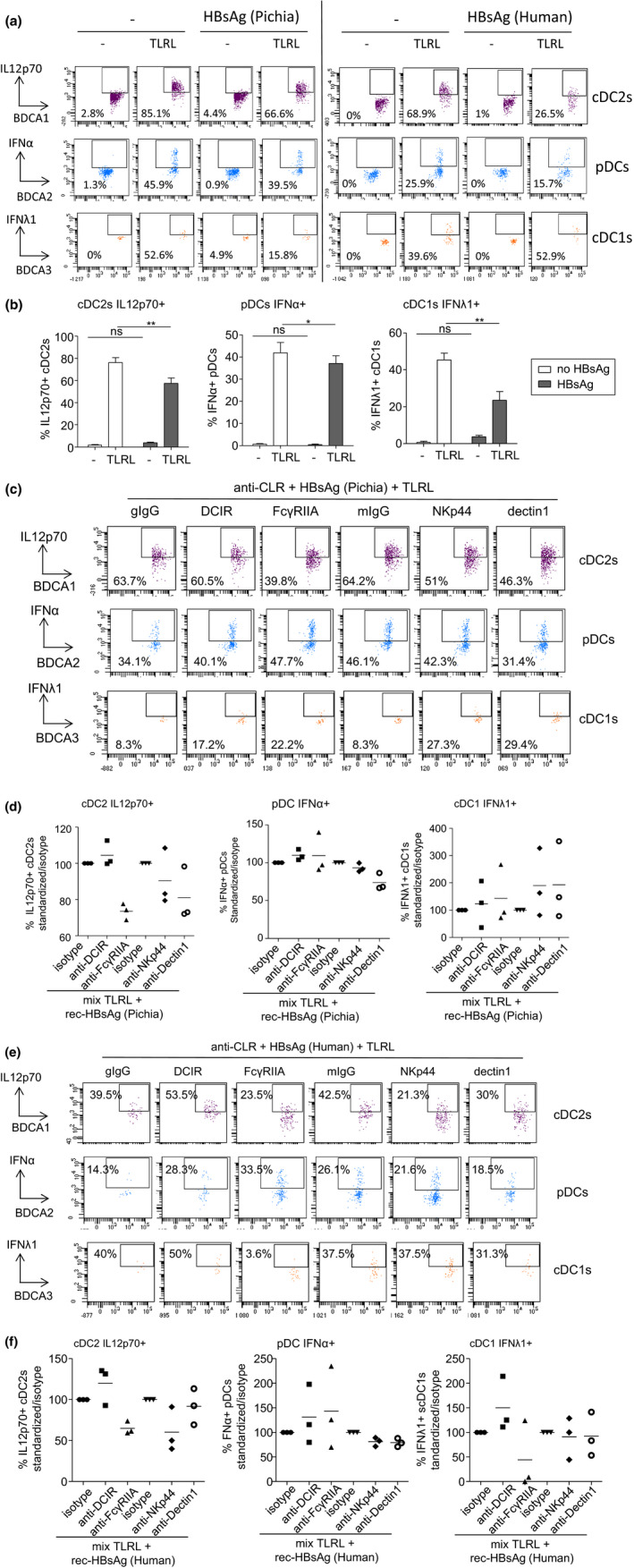
The functional ability of DC subsets to secrete cytokines in response to TLRL stimulation is modulated by HBsAg in a CLR‐dependent manner. PBMCs were cultured 30min at 37°C with or without anti‐DCIR, anti‐FcɣRIIA, anti‐DECTIN1, anti‐NKp44 blocking Abs or corresponding control isotypes. Rec‐HBsAg (produced in Pichia or Human) was added or not, and cultures were stimulated for 5 h in presence or not of a TLRL mixture (MIX: polyI:C+R848+CpGA). The productions of IL12p70 by cDC2s, IFNα by pDCs and IFNλ1 by cDC1s were evaluated by intracellular cytokine staining using flow cytometry. **(a)** Representative flow cytometry dot plots displaying intracellular stainings for IL12p70, IFNα and IFNλ1 within respectively BDCA1^+^ cDC2s (purple, top panels), BDCA2^+^ pDCs (blue, middle panels), and BDCA3^+^ cDC1s (orange, lower panels) following stimulation or not with the TLRL mixture in absence or presence of HBsAg derived from Pichia (left part) or Human (right part). **(b)** Comparative proportions of cytokine‐expressing cDC2s, pDCs and cDC1s upon stimulation or not with TLRL in presence or not of HBsAg (Pichia) (*n* = 3). Results are expressed as percentages of cytokine‐expressing cells within the corresponding DC subset. Bars indicate mean. Stars indicate significant difference with control without stimulation from each group. *P*‐values were calculated using the two‐way RM ANOVA with Bonferroni post‐tests. *P < 0.05, **P < 0.01. **(c)** Representative flow cytometry dot plots displaying intracellular stainings for IL12p70, IFNα and IFNλ1 within respectively BDCA1^+^ cDC2s, BDCA2^+^ pDCs, and BDCA3^+^ cDC1s following stimulation with the TLRL mixture in presence of HBsAg (derived from Pichia) and in the presence of Abs blocking the indicated CLRs. **(d)** Assessment of the impact of CLR blocking on the modulation of cytokine secretion by DC subsets by HBsAg (Pichia) upon TLRL stimulation (*n* = 3). Proportions of cytokine‐secreting DCs were standardised to their corresponding isotype control condition (goat IgG or mIgG2b). **(e)** Same as **c** but in the presence of HBsAg derived from Human. **(f)** Same as **d** but in the presence of HBsAg derived from Human.

**Figure 8:**
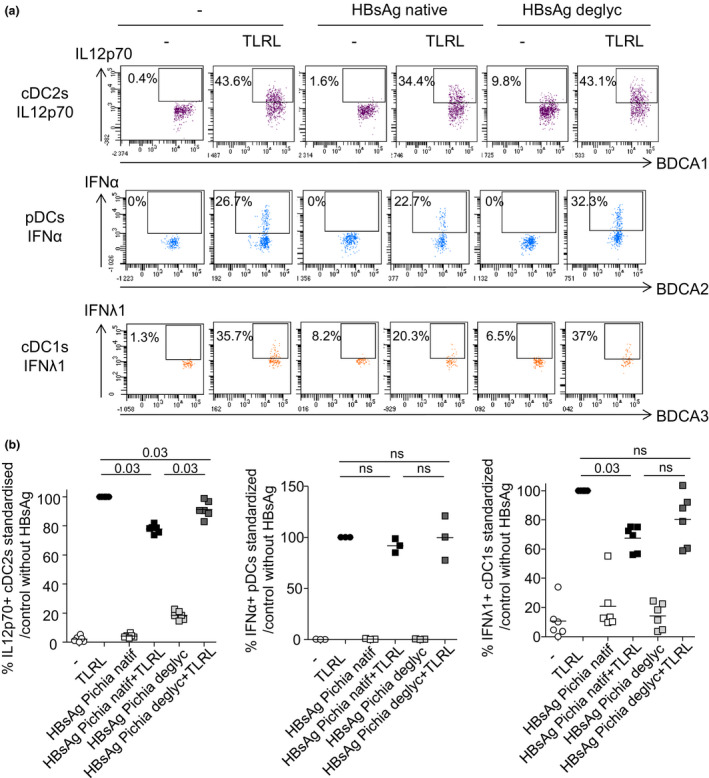
The functional inhibition of DC subsets is dependent on the glycosylation pattern of HBsAg. PBMCs were cultured in presence or not of native or deglycosylated (deglyc) HBsAg (produced in Pichia) for 5 h in presence or not of a mixture of TLRL (MIX: polyI:C+R848+CpGA). The productions of IL12p70 by cDC2s, IFNα by pDCs, and IFNλ1 by cDC1s were evaluated by intracellular cytokine staining using flow cytometry. **(a)** Representative flow cytometry dot plots displaying intracellular stainings for IL12p70, IFNα and IFNλ1 within respectively BDCA1+cDC2s (purple, top panels), BDCA2^+^ pDCs (blue, middle panels), and BDCA3^+^ cDC1s (orange, lower panels) following stimulation or not with the TLRL mixture in absence or presence of native or deglycosylated HBsAg. **(b)** Comparative proportions of cytokine‐expressing cDC2s, pDCs and cDC1s upon stimulation or not with TLR‐L in presence or not of native or deglycosylated HBsAg (*n* = 3–6). Proportions of cytokine‐secreting DCs were standardised to their corresponding control condition (TLRL without HBsAg). *P*‐values were calculated using the Wilcoxon paired *t*‐test.

**Figure 9:**
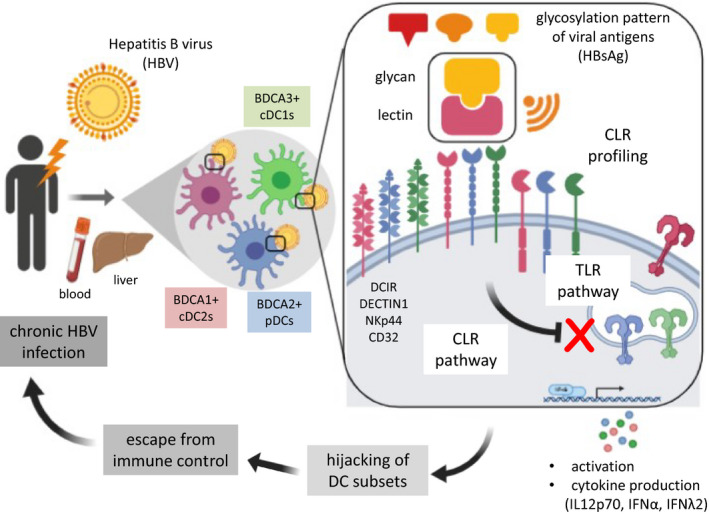
HBV exploits C‐type Lectin Receptors to hijack cDC1s, cDC2s, and pDCs and escape from immune control. Dendritic cells (DCs) are crucial in orchestrating immunity to pathogens. Sensing of virus/viral components and uptake by C‐type lectin receptors (CLRs) expressed by DCs initiate and shape antiviral immunity and are decisive in the outcome of infection. We demonstrated that the CLR repertoire of circulating and intrahepatic cDC2s, cDC1s, and pDCs is perturbed in patients with chronic HBV infection. We identified candidate CLRs responsible for HBsAg binding to cDCs (DCIR, CD32) and pDCs (DECTIN1, NKp44), and demonstrate that HBsAg inhibits DC functions in a CLR‐dependent manner. Thus, HBV exploits CLR pathways to hijack DC subsets and escape from immune control, paving the way for developing innovative antiviral approaches by manipulating the viral glycan‐lectin axis to restore an efficient immune control of the infection.

Furthermore, in order to identify the specific players involved in these cytokine secretions, we evaluated the productions of IL12p70 by cDC2s, IFNα by pDCs, and IFNλ1 by cDC1s by performing intracellular cytokine staining using flow cytometry. We confirmed that the proportions of cytokine‐expressing cDC2s, pDCs, and cDC1s upon stimulation with TLR‐L decreased in presence of HBsAg (derived from Pichia or Human) (Figure 7a and b). In addition, the productions of IL12p70 by cDC2s, IFNα by pDCs, and IFNλ1 by cDC1s were modified by the blocking of the candidate CLRs previously shown to be involved in the binding of HBsAg to DC subsets both for HBsAg derived from Pichia (Figure 7c and d) and from Human (Figure 7e and f). Notably, such functional inhibition of DC subsets was dependent on the glycosylation pattern of HBsAg, as it was abrogated upon deglycosylation of HBsAg (Figure 8). Altogether, these observations suggest that HBsAg may hijack DC functionality through specific CLRs, modulating TLR signalling pathway in a CLR‐dependent manner. By attaching to specific CLR, HBsAg might interfere with TLR pathways, exploiting the interactions between CLR and TLR pathways to impair the secretion of antiviral cytokines by DCs and escape immunity (see Figure 9).

## Discussion

Recognition of virus/viral components and uptake by CLRs together with subsequent signalling cascades are crucial to initiate and shape antiviral immunity, and decisive in the outcome of infection. Yet the interaction of HBV with CLRs remains largely unknown. Our pioneer study analysed whether HBV may exploit CLR pathways to hijack DC subsets and escape from immune control. We bring insights into the mechanisms by which HBV subverts immunity (see Graphical abstract), opening the way to develop innovative antiviral strategies exploiting glycans‐lectins interactions as targets to prevent viral recognition and/or trigger efficient antiviral immunity.

C‐type lectin receptors are key receptors used by DCs to orchestrate responses to pathogens. We highlight for the first time that the CLR repertoire of circulating and intrahepatic cDC2s, cDC1s, and pDCs is perturbed in patients with chronic HBV infection, and that some of these modulations correlated with clinical viral parameters especially HBsAg and HBV DNA levels. Major CLR modulations could be observed for DCIR for all DC subsets, DECTIN1 and MMR for cDCs, NKp44 and ILT7 on pDCs, and CLEC9A on cDC1s. Such observations indicated that HBV carbohydrates may interact with the CLR machinery to modulate DCs. As CLRs are crucial for DCs to initiate and regulate innate and adaptive immunity, HBV may influence DC polarisation and function and modify the outcome of the response by exploiting glycan‐CLR interactions. In line with our previous observations regarding the inhibition of DC subset function in HBV patients,^30^, ^35^ we demonstrate here that HBsAg binds to cDC2s, cDC1s, and pDCs from HD in a CLR‐ and glycosylation‐dependent manner, and inhibits their functional response to specific TLR triggering. Such subversion may result from the inhibition of TLR pathways by CLR signalling. Thus the exploitation of the CLR network of DCs by HBV might be a mechanism by which HBV infection progresses to chronicity.

Interestingly, the modulation of CLR expression detected in chronically infected patients revealed some opposite impact on DC subsets of the blood versus liver compartment. A possibility is that the expression of some CLRs varies according to the differentiation/maturity of the DC subsets. In a previous study^30^ in which we analysed the expression of differentiation/maturity markers of DC subsets in chronically infected HBV patients, we highlighted that cDC2s, cDC1s and pDCs from the liver compartment displayed higher levels of co‐stimulatory molecules compared to the circulating DC subsets. This differential activation status of DC subsets may explain the distinct CLR modulations observed in blood and liver. Furthermore, we previously demonstrated in chronic HBV patients dysfunctions in the capacity of circulating DC subsets to produce IL‐12p70, TNFα, IFNα, IFNλ1, and IFNλ2 upon TLR triggering.^30^ Previous reports in the context of other chronic infections such as HIV highlighted that the virus can drive an activation‐induced exhaustion of pDCs associated with an impaired functionality.^41^ HBV may affect the maturity and functionality of the DC subsets in the different compartment, thus potentially favoring transition from acute to chronic infection by limiting the efficacy of innate immunity.

Viruses such as HBV are obligate intracellular pathogens that can hijack and benefit from the host cell glycosylation machinery to modify viral proteins.^42^ Glycosylation is a diverse process that produces a repertoire of complex glycans covalently attached to proteins and lipids on the surface of viruses, relying on orchestrated action of glycosyltransferases and glycosidases generating N‐linked and O‐linked glycans. Glycosylation of viral surface proteins occurs during viral replication inside host cells. Glycan moieties of viral glycoproteins are essential for their biological functions, involved in stability and antigenicity, but also impacted the binding to host cell receptors and subsequent internalisation, infectivity and immune evasion.^43^ The glycosylation state of the pathogen hence determines its infectivity, and ability to escape immune responses. N‐glycosylation of viral surface proteins can promote proper folding and trafficking using host cellular chaperons and folding factors. Changes in glycosylation (N‐glycosylations, specific oligosaccharides) can affect interactions with receptors, the virus becoming more recognisable by the innate factors of host immune cells than by neutralising antibodies, essential for immune evasion.^43^ Virus glycosylation is crucial for viral virulence and immune escape by avoiding detection and hijacking immune interactions.^43^ Glycans confer on proteins and lipids a high diversity of structures and functions, and changes in the viral glycome may affect the virus–host interactome. Interestingly, HBsAg harbours a specific glycosylation pattern. It has been shown that HBV may escape recognition by DC‐SIGN due to branched oligosaccharides that prevent interaction between HBsAg and DC‐SIGN.^40^ Furthermore, poorly mannosylated HBV Ag escapes recognition by DC‐SIGN and lead to defective DC function. Interestingly, amino acid substitutions within HBsAg lead to different N‐glycosylation patterns, generating determinant subtypes with distinct immunogenicity.^44^, ^45^ In addition, the comparison of cohorts of HBV patients displaying or not anti‐HBs antibodies revealed that mutations in N‐glycosylation sites of HBsAg were responsible for immune escape.^46^ Hence, glycosylation pattern profile of HBV antigens especially HBsAg may affect their recognition by CLR and explain the weakness of antiviral immune response in HBV chronic infection, participating in HBV immune escape.

Potential interactions between HBsAg and specific CLRs have already been suggested: DC‐SIGN,^40^ MMR,^47^ LSECtin.^48^ Potential interactions between DC‐SIGN and HBsAg could be observed only when using glycan modified‐(highly mannosylated) HBV and not with native HBV.^40^ Interactions between HBsAg and MMR have been indirectly suggested based on positive correlations between HBsAg positivity and MMR expression level in liver and blood‐derived DCs from HBV patients.^37^ A role for LSECtin has been proposed based on LSECtin KO mouse models of viral hepatitis.^48^ In line with these reports, we also found a modulation of DC‐SIGN and MMR on DC from HBV patients but we observed that these CLRs were not able to bind HBsAg. Even though we cannot exclude that HBV/viral components interact with such CLR mostly involved in binding and internalisation, targeting the pathogen to lysosomes for degradation and cross‐presentation to T cells, we observed that HBV may preferentially exploit CLRs whose signalling inhibit function of DCs and interfere with signalling by other PRRs. Indeed, we highlight four CLRs/adaptor candidates able to bind HBsAg: DCIR, DECTIN1, NKp44, and FcɣRIIA. Through these candidate CLRs, HBV may target and subvert all three major DC subsets, therefore contributing to viral immune escape: pDCs essential for type I IFN response which is a strong innate immune defence mechanism, key in antiviral program and modulation of adaptive immunity,^8^, ^10^, ^49^, ^50^ BDCA3^+^ cDC1s crucial for cross‐presentation and CD8 T‐cell responses^51^, ^52^ and BDCA1^+^ cDC2s key for CD4 T‐cell responses.^5^, ^7^

DCIR recognises mannose/fucose‐based glycans and signals through ITIM motifs. It has been shown that cross‐linking DCIR on DCs with agonists Abs inhibited TLR8‐driven production of IL‐12 by cDCs and TLR9‐induced IFNα by pDCs and antigen presentation.^53^, ^54^ Interestingly, DCIR acts as an attachment factor for HIV‐1 on DCs and contributes to virus capture, dissemination^23^ and promotion of infection of CD4^+^ T cells.^23^ Targeting DCIR can, however, lead to antigen processing and presentation for efficient T‐cell priming.^54^, ^55^ DCIR being expressed by many cell types (DCs but also macrophages and B cells), the binding of HBsAg to DCIR may negatively impact many cell types.

DECTIN1 recognises β‐1,3‐glucans expressed by a broad range of fungal pathogens and bacteria, and also endogenous factors such as galectin‐9 or N‐glycans on tumor cells. A high signalling flexibility has been observed for DECTIN1 in response to the same ligand, potentially depending on the amino acid sequence of the CLR depending on the valency of the ligand.^56^ By cooperation with TLR2/MyD88, DECTIN1 can increase pro‐inflammatory cytokine production (IL‐12, TNFα, ROS)^57^ and activate NLRP3 inflammasome. However, DECTIN1 can protect against chronic liver disease by suppressing TLR4 signalling through reduction of TLR4 and CD14 expression.^58^ DECTIN1 can also leave an epigenetic imprinting that affects deferred signalling by heterologous receptors, a process named ‘trained immunity’ (enhanced protection to a rechallenge).^15^ Interestingly, an antigen conjugated to anti‐ DECTIN1 Abs lead to the stimulation of antigen‐specific Th17 cells.^59^ DCs facilitate the differentiation of IL17‐producing CD4 Th cells through the DECTIN1/ SYK/ CARD9 axis.^60^ Through DECTIN1, HBV may drive Th17‐oriented responses that have been shown to be elevated in HBV‐infected patients^61^, ^62^ and to participate in liver fibrosis, inflammation and HCC occurrence.^63^

NKp44 has been shown to be involved in the recognition of virally infected cells.^64^ Notably, it has been suggested that NKp44 can bind viral components such as hemagglutinins,^64^ and such interaction may further down‐regulate innate responses of pDCs. Furthermore, the cross‐linking of NKp44 (agonisation) leads to inhibition of IFNα production in response to CpG^18^. NKp44 being expressed by both pDCs and NK cells, HBV may influence these two cell types through NKp44.

We further tried to decipher the direct interactions between HBsAg and the recombinant candidate CLRs using two methods. We evaluated the capacity of HBsAg to bind to CLR‐coated polystyrene particles generated using carbodiimide‐coupling chemistry, but signals were low. We also attempted to characterise HBsAg/CLR interactions by Surface Plasmon Resonance (SPR) using previously published protocols.^65^ However, any signal with a sufficient quality was not observable and interpretable as binding of CLR over immobilised HBsAg, certainly because of the direct immobilisation strategies of HBsAg that induced a lack of functionality or damage conformation.

As glycan‐lectin interactions are pivotal in many viral infections, interfering with them or promoting recognition of viruses by specific immune system lectins are attractive strategies to counteract pathogen virulence and develop antiviral strategies.^13^, ^42^ CLRs represent promising targets for new therapies to either block viral entry into host cells preventing virus dissemination, or manipulate DC‐mediated outcome of immune responses in order to reverse viral‐induced immune evasion. Inhibition of glycan‐lectin binding could be achieved using molecules that physically interfere with these interactions such as glycan decoys (carbohydrate‐containing drugs, glycomimetics) or carbohydrate‐binding agents. Another strategy consists in altering the host/viral glycome using glycosidases/sialidases or modification of glycan synthesis. Glycome‐modifying drugs will generate aberrant glycosylation that will prevent interactions with specific lectins, interfere with assembly of virions, alter the capacity of virus to escape recognition by antibodies and cells. This field is still unexplored in HBV and worth to be better understood and exploited. Better understanding the interplay between HBV and CLR may lead to the design of new therapeutic strategies.

There are many glycan–lectin interactions during viral infections that shape virus–host interplays and are crucial for determining outcome of infection. CLRs are crucially positioned at the frontline between pathogens and innate and adaptive antiviral immune responses. Interactions between viral glycans and CLRs are essential to trigger antiviral immune responses and viral clearance, but pathogens exploit their function to evade immunity. The present work sheds light on a new pathway used by HBV to escape immunity. Better understanding glycan–lectin interactions may be harnessed to develop innovative antiviral approaches.

## Methods

### Patient and control samples

This study protocol was approved by the research ethics committee of Grenoble University Hospital (CHU‐Grenoble), the ‘Comite de Protection des Personnes’ (CPP) (no ID‐RCB: 2018‐A02164‐51) and is registered within the collection DC2008‐787. Written informed consent was obtained from all participants prior to their enrolment in the study. Blood samples were obtained from chronic HBV‐infected patients (HBV, *n* = 137) and HD (*n* = 207). PBMCs were isolated from heparinised blood samples using Ficoll‐paque density gradient centrifugation according to the manufacturer's instructions (Eurobio, Les Ulis, France). Plasma samples were collected and stored frozen. Serum HBsAg and viral load (HBV DNA) levels were quantified using the Abbott Architect i2000sr‐QT assay (Abbott, Rungis, France) and COBAS AmpliPrep/TaqMan (Roche, Boulogne‐Billancourt, France) respectively. Liver biopsy samples were obtained from chronic HBV‐infected patients (HBV, *n* = 40) and non‐viral infected control patients (CTRL, *n* = 53). Briefly, liver tissue was reduced to cell suspension (LMNCs) using a potter and the materials were subsequently separated from other aggregates using a 50 µm cell strainer. Exclusion criteria included autoimmune hepatitis, infection with human immunodeficiency virus, co‐infection with hepatitis C or D virus, and current treatment with IFNα or immunosuppressive agents. The clinical characteristics of the patients are summarised in Supplementary table 1.

### Flow cytometry analysis of basal CLR expression on DC subsets within PBMCs and LMNCs

Fresh PBMCs and LMNCs from HBV or HD/CTRL were stained with fluorochrome‐labelled anti‐human CD11c, HLA‐DR, Lineage cocktail (CD3/CD14/CD16/CD19/CD20/CD56), DNGR1/CD370/CLEC9A, CD123, MMR/CD206, DC‐SIGN/CD209 (BD Biosciences, Le Pont de Claix, France); CD45, DECTIN1/CD369/CLEC7A, CD371/CLEC12A, DEC205/CD205, Langerin/CD207, FcɛRIα; FcɣRIIA/CD32 (Biolegend, Paris, France), BDCA1/CD1c, NKp44/CD336 (Beckman Coulter, Roissy, France); BDCA2/CD303/CLEC4C, BDCA3/CD141, BDCA4/CD304 (Miltenyi Biotec, Paris, France); DCIR/CD367/CLEC4A (R&D systems, Rennes, France) and FcɣRIIA/CD32, ILT7/CD85g (eBiosiences, Paris, France) antibodies. Stained cells were then fixed with FACS lysing solution (BD) and further analysed using LSRII Flow Cytometer and FACSDiva software (BD). For PBMCs, 2 × 10^6^ CD45^+^ cells were acquired. For LMNCs, 350 000‐1 × 10^6^ CD45^+^ cells were analysed. Dead cells were excluded with Live & Dead cell stain (Thermo Fisher, Illkirch, France). The DC populations were identified as CD45^+^HLA‐DR^+^Lin‐ cells and subdivided as CD11c^+^ BDCA1^+^ cDC2s, CD11c^+^ BDCA3^+^ cDC1s, and CD11c− BDCA4^+^ CD123^+^ pDCs. Isotype controls were used to discriminate positive cells from nonspecific background staining. Both proportions and Mean Fluorescence Intensities (MFIs) were analysed. MFIs were taken on the cells positive for the corresponding markers, and shown only when proportions were above 20%. To ensure quality control during the study, we performed a standardisation of the fluorescence intensities using cytometer setup and tracking beads (BD).

### HBV antigens reagents, fluorescent labelling and deglycosylation

Recombinant HBV Surface Antigen (rec‐HBsAg), adw subtype, produced in *Pichia pastoris* or purified from human blood (as indicated on Figure legends), was purchased from Jena Biosciences (Jena, Germany) or MyBioSource (San Diego, CA, USA) respectively. Recombinant HBV Core Antigen (rec‐HBcAg) produced in *P. pastoris*, was purchased from Meridian Life Sciences (Cincinnati, OH, USA). It is worth noting that this rec‐HBsAg is only composed of the small envelope protein (S) and neither contains L nor M envelope proteins. We carefully selected productions with the highest purity assessed by RP‐HPLC and SDS‐PAGE (purity > 95%). For some experiments, rec‐HBsAg and rec‐HBcAg were conjugated to DyLight 650 sulfhydryl‐reactive dye according to manufacturer instructions (Thermo Fisher Scientific). After conjugation, unbound conjugate was removed by fluorescent Dye Removal columns (Thermo Fisher Scientific) according to manufacturer instructions. Fluorescent labelled‐HBsAg (FL‐HBsAg) and fluorescent labelled‐HBcAg (FL‐HBcAg) were then stored at −20°C for *in vitro* uptake/binding experiments. The quality of labelling in a dye‐in‐solution sample (FL‐HBsAg and FL‐HBcAg) was systematically assessed by multiparametric confocal microscopy on LSM710 NLO – LIVE7 – Confocor3/ Dynascope (Carl Zeiss, Oberkochen, Germany) and the fluorescence correlation spectroscopy data were acquired and analysed. The count rate (i.e. amount of fluorescence coming back from the sample based on the beam path) and the counts per second per molecule (i.e. direct measure of the amount of fluorescence per molecule) were evaluated and the interaction/labelling between the fluorochrome (maleimide 650 sulfhydryl‐reactive dye reactive) and the rec‐HBsAg or rec‐HBcAg proteins were measured at a single molecule level (Carl Zeiss). For some experiments, rec‐HBsAg from Pichia or Human was deglycosylated using the Glycoprotein Deglycosylation Kit (Sigma, Lezennes, France) according to manufacturer' instructions. We performed the non‐denaturing protocol that allows the enzymatic removal of N‐/O‐linked oligosaccharides (N‐glycosidase‐F, acetylgalactosaminidase, neuraminidase, acetylglucosaminidase, galactosidase).

### Exposure of PBMCs and purified pDCs to HBsAg

To analyse the impact of rec‐HBsAg exposure on CLR expression on DCs, total PBMCs or purified pDCs (StemCell, Grenoble, France) were cultured respectively at 1 × 10^6^ cells mL ^−1^ or 4 × 10^5^ cells mL^−1^ for 4 h at 37°C in 48‐wells plate or 96‐round bottom well plates (Corning, Wiesbaden, Germany) in presence or not of rec‐HBsAg (Jena Biosciences) together or not with Class‐A CpG oligonucleotide ODN‐2336 (CpG_A_, 1 µm) (Invivogen, Toulouse, France). All cultures were performed in RPMI‐1640/GlutaMAX (Invitrogen, Courtaboeuf, France) supplemented with 1% non‐essential amino acids, 100 µg mL^−1^ gentamicin, 1 mm sodium pyruvate (Sigma), and 10% foetal bovine serum (FBS) (Invitrogen) or autologous human serum. Subsequently, cells were thoroughly washed with cold PBS supplemented with 2% of FBS to remove the free HBsAg particle and then, cells were stained with fluorochrome‐labelled anti‐human CD11c, HLA‐DR, Lineage cocktail, DNGR1/CD370/CLEC9A, CD123, MMR/CD206, DC‐SIGN/CD209 (BD); Lineage cocktail, CD45, DECTIN1/CD369/CLEC7A, CD371/CLEC12A, DEC205/CD205, Langerin/CD207, FcɛRIα; (Biolegend), BDCA1/CD1c, NKp44/CD336 (Beckman Coulter); BDCA2/CD303/CLEC4C, BDCA3/CD141, BDCA4/CD304 (Miltenyi Biotec); DCIR/CD367/CLEC4A (R&D systems), and FcɣRIIA/CD32, ILT7/CD85g (eBiosciences) antibodies. The analysis of CLRs expression after HBsAg exposure was performed using LSRII Flow Cytometer and FACSDiva software (BD).

### Uptake/binding of HBsAg/HBcAg by DC subsets *in vitro*

To highlight HBsAg uptake/binding on DC subsets, PBMCs were seeded at 1 × 10^6^ cells mL^−1^ in 48‐well plates (Corning) and exposed for 2 h at 4 or 37°C to 25 µg mL^−1^ of either native or deglycosylated FL‐HBsAg or FL‐HBcAg in presence or not of a mixture of TLR ligands comprising polyI:C (30 µg mL^−1^), Imiquimod (R848, 1 µg mL^−1^), and Class‐A CpG oligonucleotide ODN‐2336 (CpG_A_, 1 µm; Invivogen). In some experiments, PBMCs were exposed to increased amounts of FL‐HBsAg from 10 to 75 µg mL^−1^. After exposure, PBMCs were thoroughly washed with cold PBS2% FBS to remove the unbound particles and cells were then stained for CD11c, HLA‐DR, Lineage cocktail, CLEC9A, CD123 (BD); CD45 (Biolegend), and BDCA1 (Beckman Coulter) antibodies and further fixed with FACS lysing solution (BD). FL‐HBsAg‐positive and FL‐HBcAg‐positive cDC1s, cDC2s and pDCs were then analysed using LSRII Flow Cytometer and FACSDiva software (BD).

### HBsAg uptake/binding assays upon CLR neutralisation

To determine the involvement of specific CLRs as putative attachment factors for HBsAg on DCs, we performed neutralisation experiments by blocking single CLR using specific functional grade anti‐human CLR antibodies. Briefly, PBMCs were seeded at 2 × 10^6^ cells mL^−1^ in 48‐well plates (Corning) and pre‐incubated for 30 min at 37°C in presence or not of blocking anti‐human DCIR, FcɣRIIA, MMR, FcεRIα, DECTIN1, BDCA2 (R&D systems), NKp44 (Invitrogen and Biolegend), ILT7 (Invitrogen) antibodies (single or in combination) or specific isotype controls. After 30 min of CLR blockade, 25 µg mL^−1^ of FL‐HBsAg was added in the culture for an additional 2 h at 37°C. Subsequently, PBMCs were thoroughly washed with cold PBS2% FBS to remove solution‐free anti‐human CLR antibodies or FL‐HBsAg. Hence, cells were stained with anti‐CD11c, HLA‐DR, Lineage cocktail, CLEC9A, CD123, (BD), CD45 (Biolegend) and BDCA1 (Beckman Coulter) antibodies and fixed with FACS lysing solution (BD). FL‐HBsAg‐positive cDC1s, cDC2s and pDCs in each condition were then analysed using LSRII Flow Cytometer and FACSDiva software (BD).

### Cytokine secretion upon TLRL stimulation after CLR neutralisation

To determine the impact of blocking CLR on DCs function upon stimulation with mixed TLRL in presence of HBsAg, we performed experiments consisting in blocking single CLR in presence or not of rec‐HBsAg and TLRL stimulation. Briefly, PBMCs were seeded at 1 × 10^6^ cells mL^−1^ in 96‐well plates (Corning) and cultured for 30 min at 37°C in presence or not of blocking anti‐human FcɣRIIA, FcεRIα, DCIR, BDCA2 antibody, DECTIN1, MMR (R&D systems), NKp44 (Invitrogen and Biolegend), ILT7 (Invitrogen) functional grade antibodies or specific isotype controls. After CLR blocking, rec‐HBsAg (from Pichia or Human) was added or not in the culture in presence or not of a mixture of TLR ligands comprising polyI:C (30 µg mL^−1^), Imiquimod (R848, 1 µg mL^−1^), and Class‐A CpG oligonucleotide ODN‐2336 (CpG_A_, 1 µm; Invivogen) and the cells were subsequently cultured for another 20 h at 37°C. (1) Cultures consisting of PBMCs in addition to anti‐human CLR antibodies allow determining the single impact of each anti‐human CLR antibody on the basal cytokine secretion. (2) Cultures involving PBMCs together with rec‐HBsAg in addition with anti‐human CLR antibodies allow determining the combined effect of each anti‐human CLR antibody in presence of rec‐HBsAg on the basal cytokine secretion. (3) Cultures consisting of PBMCs in presence of anti‐human CLR antibodies and stimulation with mixed TLRL allow determining the single impact of each anti‐human CLR antibody on the cytokine secretion after TLRL triggering. (4) Cultures consisting of PBMCs together with rec‐HBsAg in addition to anti‐human CLR antibodies and stimulation with a mixture of TLRL allow determining the combined effect of each anti‐human CLR antibody and rec‐HBsAg on the immune secretory activity of DC subsets upon TLRL triggering. Culture supernatants were harvested and IFNα2, IFNβ, IL‐12p70, IFNλ1, and IFNλ2 secretions were measured by Luminex Technology according to manufacturer protocol using MAGPIX®200 Instrument with xPONENT® software (Bio‐Rad, Cressier, Switzerland). Inter‐assay variability was determined by quantifying the same control sample each time, which was frozen in multiple aliquots thawed once the day of each assay. The basal percentage and absolute number of BDCA1^+^ cDC2s, BDCA3^+^ cDC1s, and BDCA2^+^ pDCs were systematically evaluated before the culture by labelling PBMCs with CD11c, HLA‐DR, Lineage cocktail, CLEC9A, CD123 (BD), CD45 (Biolegend), and BDCA1 (Beckman Coulter) followed by analysis using LSRII Flow Cytometer and FACSDiva software (BD).

### Intracellular cytokine staining within DC subsets in response to TLR triggering

Peripheral blood mononuclear cells were seeded at 1 × 10^6^ cells mL^−1^ in 96‐well plates (Corning) and cultured for 30 min at 37°C in presence or not of blocking anti‐human FcɣRIIA, FcεRIα, DCIR, BDCA2 antibody, DECTIN1, MMR (R&D systems), NKp44 (Invitrogen and Biolegend), ILT7 (Invitrogen) functional grade antibodies or specific isotype controls. Native or deglycosylated rec‐HBsAg (from Pichia or Human) were added or not in the culture and the cells were subsequently cultured for 5 h in presence or not of a mixture of TLR ligands comprising polyI:C (30 µg mL^–1^), Imiquimod (R848, 1 µg mL^−1^), and Class‐A CpG oligonucleotide ODN‐2336 (CpGA, 1 µm) (Invivogen). 1 µg mL^−1^ of Brefeldin A (BD) was added for the last 4 h. Later on, cells were stained for surface markers allowing to define cDC1s, cDC2s and pDCs (CD11c, HLA‐DR [BD], Lin, CD45 [Biolegend], cDC1/BDCA1 [Beckman], BDCA2 and BDCA3 [Miltenyi]) and then fixed and permeabilised according to manufacturer' instructions (BD Biosciences). Intracellular cytokine staining was then performed using the fluorochrome‐labelled anti‐human TNF⍺, IL‐12p40/70 (BD), IFN⍺ (Miltenyi) antibodies and anti‐human IFNλ1 (Novus, Abingdon, UK) antibody stained with mix‐n‐stain CF488 (Biotium, Fremont, CA, USA). Analyses were done by flow cytometry using LSRII Flow Cytometer and FACSDiva software v.8.

### Statistical analysis

Statistical analyses were performed using the Mann–Whitney non‐parametric *U‐*test, the Wilcoxon paired *t*‐test combined with Bonferroni correction, and the Spearman correlation using GraphPad Prism software version 5.01 (GraphPad, San Diego, CA, USA).

## Author Contributions

**Laurissa Ouaguia:** Conceptualization; Data curation; Formal analysis; Investigation; Methodology; Writing‐original draft; Writing‐review & editing. **Tania Dufeu‐Duchesne:** Data curation; Formal analysis. **Vincent Leroy:** Resources; Validation; Writing‐review & editing. **Thomas Decaens:** Resources; Validation; Writing‐review & editing. **Jean‐Baptiste Reiser:** Data curation; Formal analysis; Methodology; Resources; Writing‐review & editing. **Eleonora Sosa Cuevas:** Formal analysis; Methodology. **David Durantel:** Formal analysis; Funding acquisition; Methodology; Resources; Validation; Writing‐review & editing. **Jenny Valladeau‐guilemond:** Conceptualization; Methodology; Validation; Writing‐review & editing. **Nathalie Bendriss‐Vermare:** Conceptualization; Formal analysis; Funding acquisition; Methodology; Validation; Writing‐review & editing. **Laurence Chaperot:** Formal analysis; Funding acquisition; Project administration; Validation; Writing‐review & editing. **Caroline Aspord:** Conceptualization; Data curation; Formal analysis; Funding acquisition; Investigation; Methodology; Project administration; Supervision; Writing‐original draft; Writing‐review & editing.

## Conflict of interest

The authors declare no conflict of interest.
