# Supplementary material for: Hepatitis B virus exploits C‐type lectin receptors to hijack cDC1s, cDC2s and pDCs
Source: Clin Transl Immunology. 2020 Dec 8;9(12):e1208. doi: 10.1002/cti2.1208 (PMC7723857; doi:10.1002/cti2.1208)

Supporting Information

Supplementary Table 1: Clinical characteristics of the patients included in the study

Supplementary Table 2: Correlations between CLR expression on circulating DC subsets and clinical parameters in HBV patients

The frequency and the basal expression of CLR molecules were evaluated within BDCA1+ cDC2, BDCA3+ cDC1 and BDCA4+ pDCs from fresh PBMCs isolated from HBV patients and correlated with the clinical viral parameters (HBsAg and HBV DNA levels) as well as with liver parameters (ALT levels). Spearman's correlations between the DCs features and HBV DNA (n=17-19) and/or plasmatic HBsAg levels (n=27-28) and/or ALT levels (n=28). Only correlations with $r \geq 0.5$ or $r \leq -0.5$ and $P \leq 0.05$, and $0.4 \leq r \leq 0.5$ or $-0.5 \leq r \leq -0.4$ with $P \leq 0.03$ are notified. Blue: negative correlation; Red: positive correlation. *$P \leq 0.05$, **$P \leq 0.01$, *** $P \leq 0.001$.

Supplementary Table 3: Correlations between CLR expression on intrahepatic DC subsets and clinical parameters in HBV patients

The frequency and the basal expression of CLR molecules were evaluated within BDCA1+ cDC2, BDCA3+ cDC1 and BDCA4+ pDCs from fresh LMNCs from HBV patients and correlated with the clinical viral parameters (HBsAg and HBV DNA levels) as well as with liver parameters (ALT levels). Spearman's correlations between the DCs features and HBV DNA (n=8-13) and/or plasmatic HBsAg levels (n=7-13) and/or ALT levels (n=9-13). Only correlations with $r \geq 0.5$ or $r \leq -0.5$ with $P \leq 0.05$ are notified. Blue: negative correlation; Red: positive correlation. *$P \leq 0.05$, **$P \leq 0.01$, ***$P \leq 0.001$.

Supplementary Figure 1. Gating strategy for circulating and intrahepatic DC subsets from chronic HBV patients and analysis of CLR expression

(A) DC subsets gating strategy (representative flow cytometry plots of HBV patients). Fresh PBMC or LMNC suspensions were pre-gated on single alive CD45+ cells. The DC populations were further identified as CD45+HLA-DR+Lin^{neg} (Lin: CD3/CD14/CD16/CD19/CD20/CD56) cells and subdivided as CD11c+ BDCA1+ cDC2s, CD11c+ BDCA3+ cDC1s, and BDCA4+ CD11c- CD123+ pDCs. **(B-D)** Analysis of CLR expression on each specific DC subset in blood and liver biopsy. Representative flow cytometry plots of DECTIN1, DCIR and MMR expression on cDC2s (B), DECTIN1,

CLEC9A and FcγRIIA expression on cDC1s (C) and DCIR, ILT7, NKp44 and FcεRIA expression on pDCs (D) from an HBV patient.

Supplementary Figure 2. Frequencies of circulating and intrahepatic DC subset's and basal CLR expression on DCs from chronic HBV patients compared to controls

(A) DC subset's frequencies (cDC2s, cDC1s, and pDCs) were analyzed by flow cytometry on peripheral blood (upper panels) and liver biopsies (lower panels) of chronic HBV patients and HD or non-viral infected controls. Results are expressed as percentages of DCs among CD45+ cells. Bars indicate median. Blood: open symbols, HD (n=27-31) and filled symbols, HBV patients (n=31-80); liver: open symbols, controls (n=23-36) and filled symbols, HBV patients (n=13-17)**.** P-values were calculated using the Mann-Whitney test. **(B-C)** CLR expression on **(B)** circulating cDC1s (FcγRIIA) and **(C)** intrahepatic cDC2s and cDC1s (DCIR, MMR). Results are expressed as MFI of positive cells. Bars indicate median. Blood: open symbols, HD (n=12-24) and filled symbols, HBV patients (n=29-32); liver: open symbols, controls (n=16-18) and filled symbols, HBV patients (n=9-12)**.** *P*-values were calculated using the Mann-Whitney test.

Supplementary Figure 3. Modulation of the basal CLR expression on pDCs from chronic HBV patients

The basal expression of CLR on pDCs was assessed from PBMCs or LMNCs from chronic HBV patients by flow cytometry. **(A)** ILT7 and NKp44 expression (MFI) on circulating pDCs. **(B)** FcεRIα expression (MFI) on intrahepatic pDCs. Results are expressed as MFI of positive cells. Bars indicate median. Blood: open symbols, HD (n=15-16) and filled symbols, HBV patients (n=43); liver: open symbols, controls (n=10) and filled symbols, HBV patients (n=7)**.** *P*-values were calculated using the Mann–Whitney test. **(C)** Spearman's correlation of FcεRIα expression (MFI) on intrahepatic pDCs with HBsAg levels (n=9).

Supplementary Figure 4. Correlations of CLR expression with clinical and liver parameters in chronic HBV patients

The basal expression of CLR on DC subsets was assessed from PBMCs and LMNCs from chronic HBV patients by flow cytometry and correlations with clinical parameters were analyzed. **(A)** Spearman's correlation of DCIR expression (MFI) on circulating cDC2s with HBV DNA levels (n=17). **(B)** Spearman's correlations of FcγRIIA or MMR expression (%/MFI) on circulating cDC1s with HBsAg levels (n=28-30), and DECTIN1 or DEC205

(%/MFI) on circulating cDC1s with HBV DNA levels (n=16-19). **(C)** Spearman's correlations of DCIR and DECTIN1 expression (% or MFI) on intrahepatic cDC2s with HBV DNA levels (n=10-13). **(D)** Spearman's correlations of DCIR, DECTIN1, FcɣRIIA, and MMR expression (%/MFI) on intrahepatic cDC1s with HBsAg levels (n=6-8), HBV DNA levels (n=8-11), or ALT levels (n=11).

Supplementary Figure 5. Expression of the disrupted CLRs on circulating DC subsets depending on plasmatic HBsAg level

The expression of CLR molecules by cDCs and pDCs was analyzed by flow cytometry from fresh PBMCs derived from chronic HBV patients according to their HBsAg levels. (**A**) Expression levels of DECTIN1, DC-SIGN, MMR on circulating cDC2s. (**B**) Expression levels of DECTIN1, CLEC9A and FcɣRIIA on circulating cDC1s. **(C)** Expression levels of DCIR, ILT7, NKp44 and FcεRIα on circulating pDCs. Bars indicate median. White squares: HBsAg<1000 IU/ml (n=6-15); light grey squares: 1.000<HBsAg<10.000 IU/ml (n=16-28); dark grey squares: HBsAg>10.000 IU/ml (n=10-26). P-values were calculated using the Mann–Whitney test (dotted lines) and Kruskal-Wallis test (full lines).

Supplementary Figure 6. Impact of HBsAg on CLR expression on DC subsets

(A-B) Purified pDCs or PBMCs were cultured for 4 hours in presence or not of rec-HBsAg (25µg/mL) and the modulation of CLR expression on each DC subset was measured by flow cytometry. Impact of rec-HBsAg on **(A)** FcɣRIIA expression on cDC1s, **(B)** NKp44 and BDCA2 expression on pDCs. Results are expressed as percentages within the corresponding subset or MFI of positive cells. Bars indicate median. Open symbols, media (n=10-17) and filled symbols, rec-HBsAg (n=10-17). P-values were calculated using the Wilcoxon paired t-test. **(C)** Purified pDCs were cultured for 4 hours in presence or not of rec-HBsAg together or not of CpG_A and CLR expression was measured by flow cytometry: (DCIR, ILT7, NKp44, FcεRIα, FcɣRIIA and BDCA2). Results are expressed as percentages of positive cells. Bars indicate mean. Open symbols, media (n=6-13); light grey symbols, rec-HBsAg 5µg/mL (n=4), dark grey symbols, rec-HBsAg 25µg/mL (n=6-14). *P*-values were calculated using the Wilcoxon paired t-test.

Supplementary Figure 7. Representative dot plots for evaluation of the binding of FL-HBsAg on cDC2s, cDC1s, and pDCs in a dose-dependent manner

PBMCs were cultured for 2 hours at 37°C in presence or not of FL-HBsAg at 10µg/mL, 25µg/mL, and 75µg/mL. The binding/fixation of FL-HBsAg on DC subsets and lymphocytes was then measured by flow cytometry. Dotplots were pre-gated on single alive CD45+, HLA-DR+, Lin^{neg} cells, and the DC populations were further subdivided as CD11c+ BDCA1+ cDC2s, CD11c+ BDCA3+ cDC1s, and BDCA4+ CD11c- CD123+ pDCs. Representative dot plots of 3 independent experiments.

Supplementary Figure 8. Impact of FL-HBsAg on cell viability and binding of monocytes

Rec-HBsAg was labeled with DyLight 650 sulfhydryl-reactive dye. PBMCs were cultured for 2 hours at 37°C in presence or not of FL-HBsAg at various concentrations. **(A)** The viability of the CD45+ cells was assessed with Live & Dead labeling. **(B)** The binding/fixation of FL-HBV-Ag on monocytes was measured by flow cytometry. Results are expressed as % of FL-HBsAg positive cells; n=3.

Supplementary Figure 9. Impact of CLR blocking on IFNα2, IL12p70, and IFNλ1/IL-29 secretions in response to TLRL stimulation

PBMCs were cultured 30min at 37°C with or without anti-BDCA2 anti- DCIR, anti-FcɣRIIA, anti-MMR, anti-FcεRIα, anti-DECTIN1, anti-NKp44, anti-ILT7 and anti-CLEC9A blocking Abs. Cultures were further incubated for 20 hours in presence of a mixture of TLRL (MIX: polyI:C+R848+CpG_A). Supernatants were then examined for the presence of IFNα2, IL-12p70, and IFNλ1/IL-29 by Luminex (n=10-11). Secretions were standardized to the corresponding isotype control condition. Bars indicate mean. *P*-values were calculated using the Wilcoxon paired t-test. *p≤0.05, **p≤0.01. Only significant statistics are shown.

Supplementary Table 1:
Clinical characteristics of the patients included in the study

	Blood samples						Liver biopsy samples	
	HBeAg positive			HBeAg negative				
**Classification**	Chronic infection	Chronic hepatitis		Chronic infection	Chronic hepatitis		Chronic Hepatitis B	Non viral liver diseases[f]
**Old terminology**	Immune tolerant	Immune reactive		Inactive carriers	HBeAg negative chronic hepatitis			
		Untreated	Treated		Untreated	Treated		
**n**	2	2	11	43	19	60	40	53
**Age (y)**	28.50 ± 13.51	25.53 ± 1.20	43.48 ± 17.62	39.16 ± 15.62	36.80 ± 14.56	54.28 ± 16.26	36.08 ± 13.8	57.58 ± 10.19
**Sex (%, Male/Female)**	50/50	100/0	90.91/9.09	65.12/34.88	63.16/36.84	73.33/26.66	75/25	58.49/41.51
**ALT level (IU/L) (ULN=35)**	90.5 ± 68.59	65.33 ± 58.60	46.64 ± 16	37.24 ± 16.53	48.05 ± 31.24	38.78 ± 18.29	64.65 ± 77.7	78 ± 46.36
**HBV DNA (log10 IU/mL)**	8.23 ± 0	6 ± 1.48	1.18 ± 0.12	2.52 ± 0.66	4.33 ± 1.36	1.20 ± 0.21	4.42 ± 1.78	-
**HBsAg (log10 IU/mL)**	4.57e	4.21 ± 0.20	3.44 ± 1.13	3.58 ± 0.90	3.85 ± 0.44	3.13 ± 0.78	3.88 ± 0.54	-
**On treatment (%)**	0[a]	0[a]	100[b]	0[a]	0[a]	100[c]	7.5[d]	-
**METAVIR A**	3[e]	1[e]	1.67 ± 0.87[e]	0.91 ± 0.70[e]	0.88 ± 0.64[e]	1.25 ± 0.73[e]	1.03 ± 0.77[e]	2.25 ± 1.30[e]
**METAVIR F**	0[e]	1[e]	1.39 ± 0.93[e]	1 ± 0.91[e]	0.80 ± 0.42[e]	1.77 ± 1.07[e]	1.29 ± 1.073[e]	1.49 ± 1.45[e]
**F3F4 (%)**	-	-	9.09[e]	2.33[e]	-	18.33[e]	12.82[e]	20.76[e]

Data are expressed as mean ± SD unless indicated otherwise
a) No treatment (100%)
b) Tenofovir (100%)
c) 3,33% Entecavir+Tenofovir, 63,35% Tenofovir, 26,66% Entecavir, 5% Lamavudine, 1,66% Adefovir
d) 7,69% treated (2 Entencavir, 1 Viread), 92,31% untreated
e) Values calculated on patients with known HBsAg value or METAVIR A and F Score
f) 40 nonalcoholic steatohepatitis, 2 mixed alcoholic and metabolic steatohepatitis, 5 unknown hypertransaminasemia, 1 hepatosiderosis, 1 anicteric cholestasis, 1 primary sclerosing cholangitis, 1 primary biliary cholangitis, 1 granulomateous hepatitis and 1 unknown steatopathy

Abreviations: ALT, alanine aminotransferase; F, female; HBeAg, hepatitis B e antigen; HBsAg, hepatitis B s antigen; HBV, hepatitis B virus; M, male ; n, number of patient; y, year; %, percentage

Supplementary Table 2:
Correlations between CLR expression on circulating DC subsets and clinical parameters in HBV patients

Circulating DC subsets		Viral patterns		Liver Patterns
		HBsAg IU/mL	HBV DNA IU/mL on viremic HBV patients	ALT IU/L
**CLR molecules on cDC2**	MFI DCIR		* (0.46)	
**CLR molecules on cDC1**	% frequency	**(0.44)		* (0.38)
	% DECTIN 1	* (-0.4)	** (-0.55)	
	% FcγRIIA	* (-0.45)		* (-0.43)
	% DEC205	* (0.43)		* (0.42)
	MFI DEC205		*** (-0.67)	
	MFI MMR	* (-0.45)		

Spearman correlation (stars indicate P, (x) indicate r)

r≥0.5 or r≤- 0.5 with *P* ≤.05, 0.4≤ r ≤0.5 or -0.5≤ r ≤-0.4 with *P* ≤.03

Blue : negative correlation

Red : positive correlation

**P* ≤0.05, ***P* ≤0.01 , ****P* ≤0.001

Supplementary Table 3:
Correlations between CLR expression on intrahepatic DC subsets and clinical parameters in HBV patients

intrahepatic DC subsets		Viral patterns		Liver Patterns
		HBsAg IU/mL	HBV DNA IU/mL on viremic HBV patients	ALT IU/L
CLR molecules on cDC2	% DCIR	* (-0.67)	* (-0.68)	
	% DECTIN 1		* (-0.65)	
	MFI DECTIN 1		* (-0.64)	
CLR molecules on cDC1	% DCIR		* (-0.75)	
	% CLEC12A	* (-0.8)		
	% DECTIN 1			** (0.72)
	% FcγRIIA	* (-0.76)		
	MFI MMR	** (-0.8)	*** (-0.89)	
CLR molecules on pDCs	MFI NKp44		* (-0.7)	
	% FcγRIIA	* (-0.55)		
	% FcεRIα	* (-0.77)		
	MFI FcεRIα	* (-0.66)	* (-0.7)	

Spearman correlation (stars indicate P, (x) indicate r)

r≥0.5 or r≤- 0.5 with *P* ≤.05

Blue : negative correlation

Red : positive correlation

**P* ≤0.05, ***P* ≤0.01 , ****P* ≤0.001

Supplementary Figure 1

a

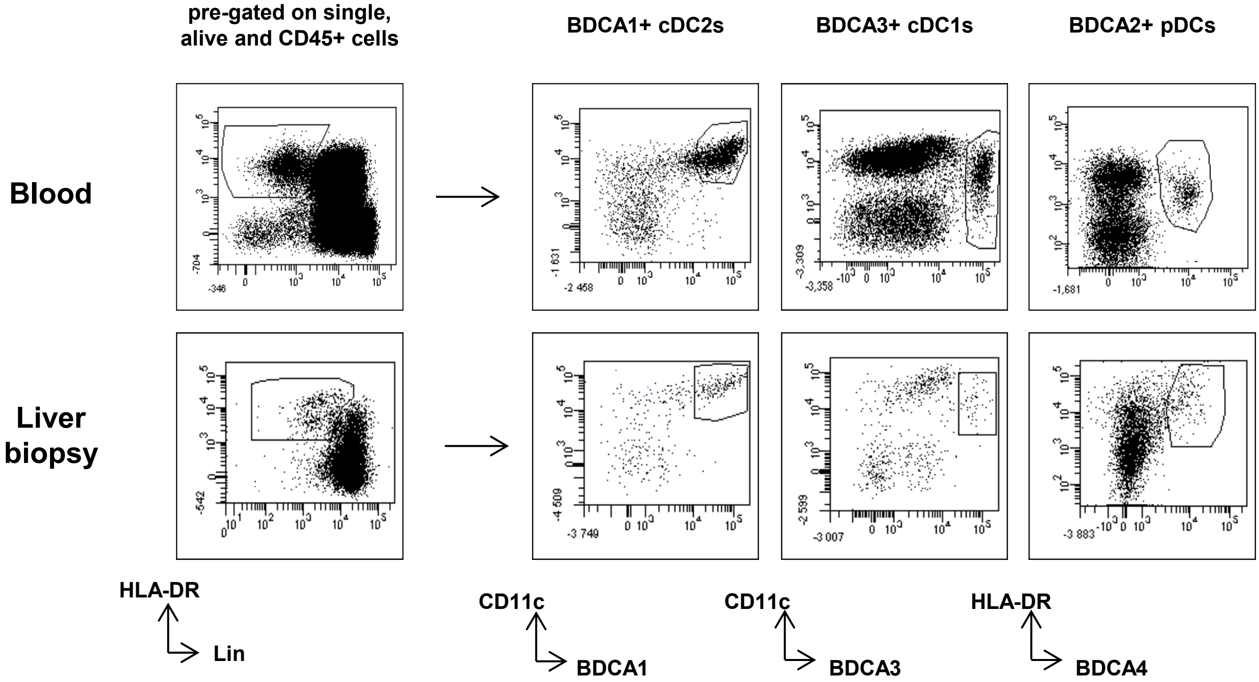

b

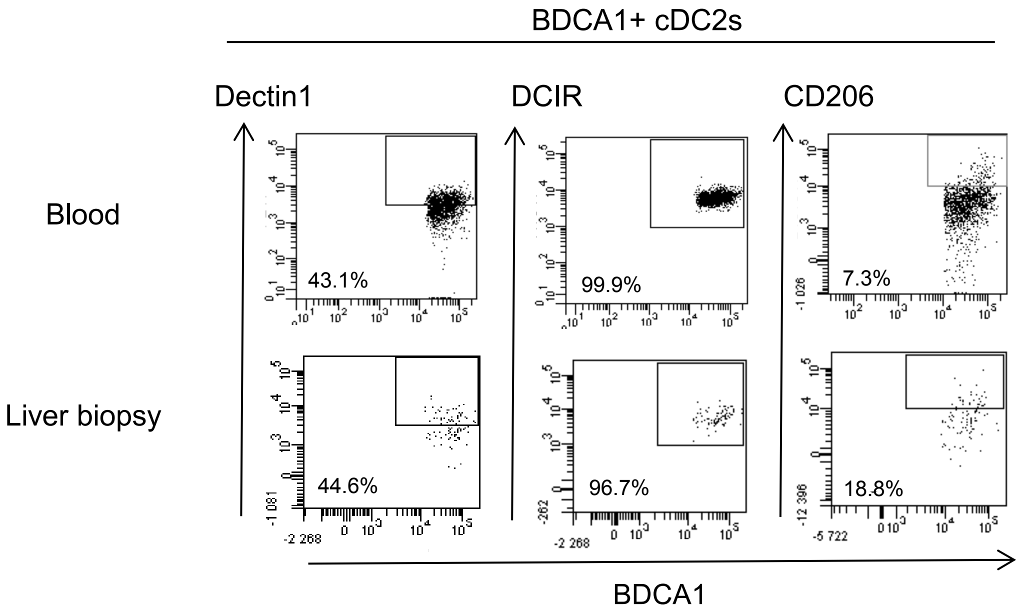

Supplementary Figure 1 (follow)

c

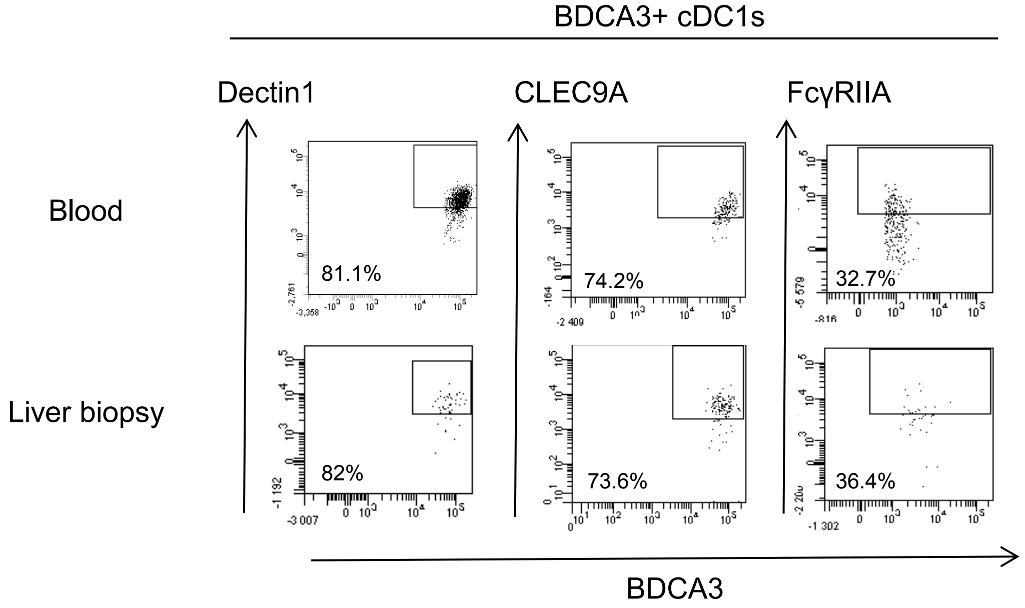

d

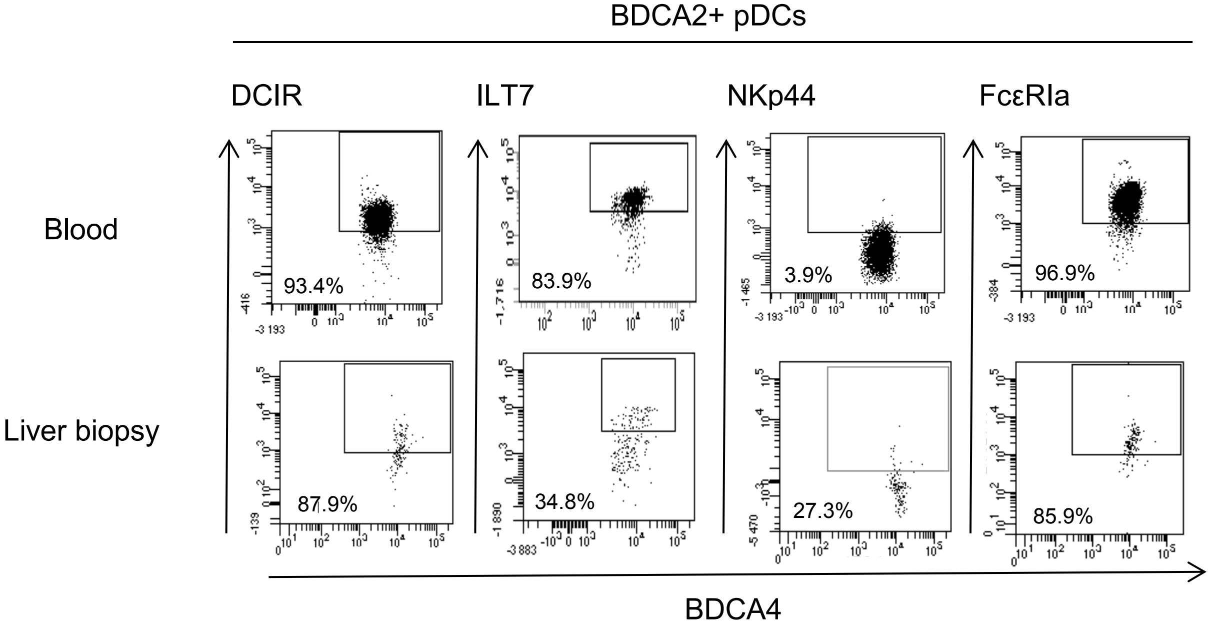

Supplementary Figure 2

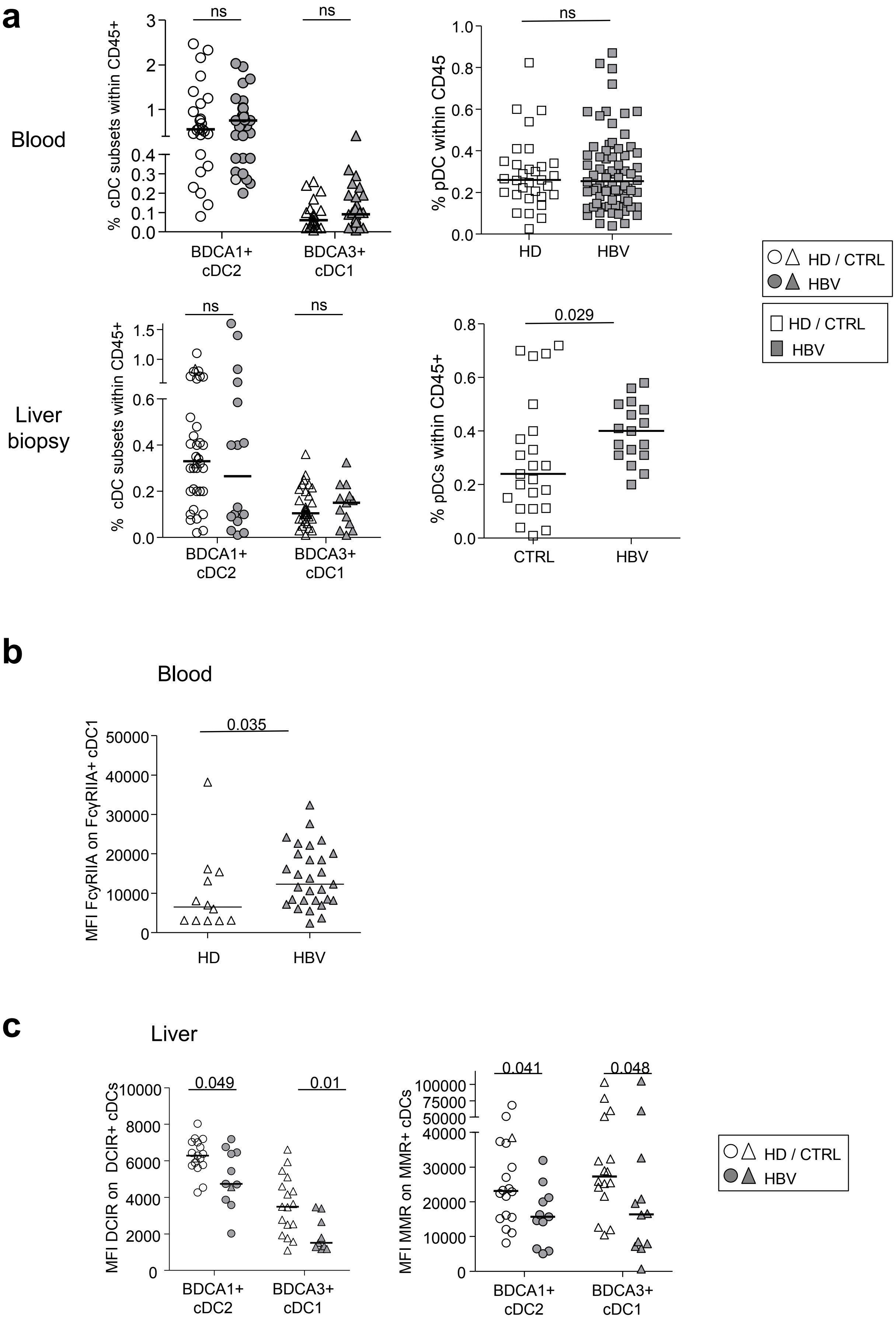

Supplementary Figure 3

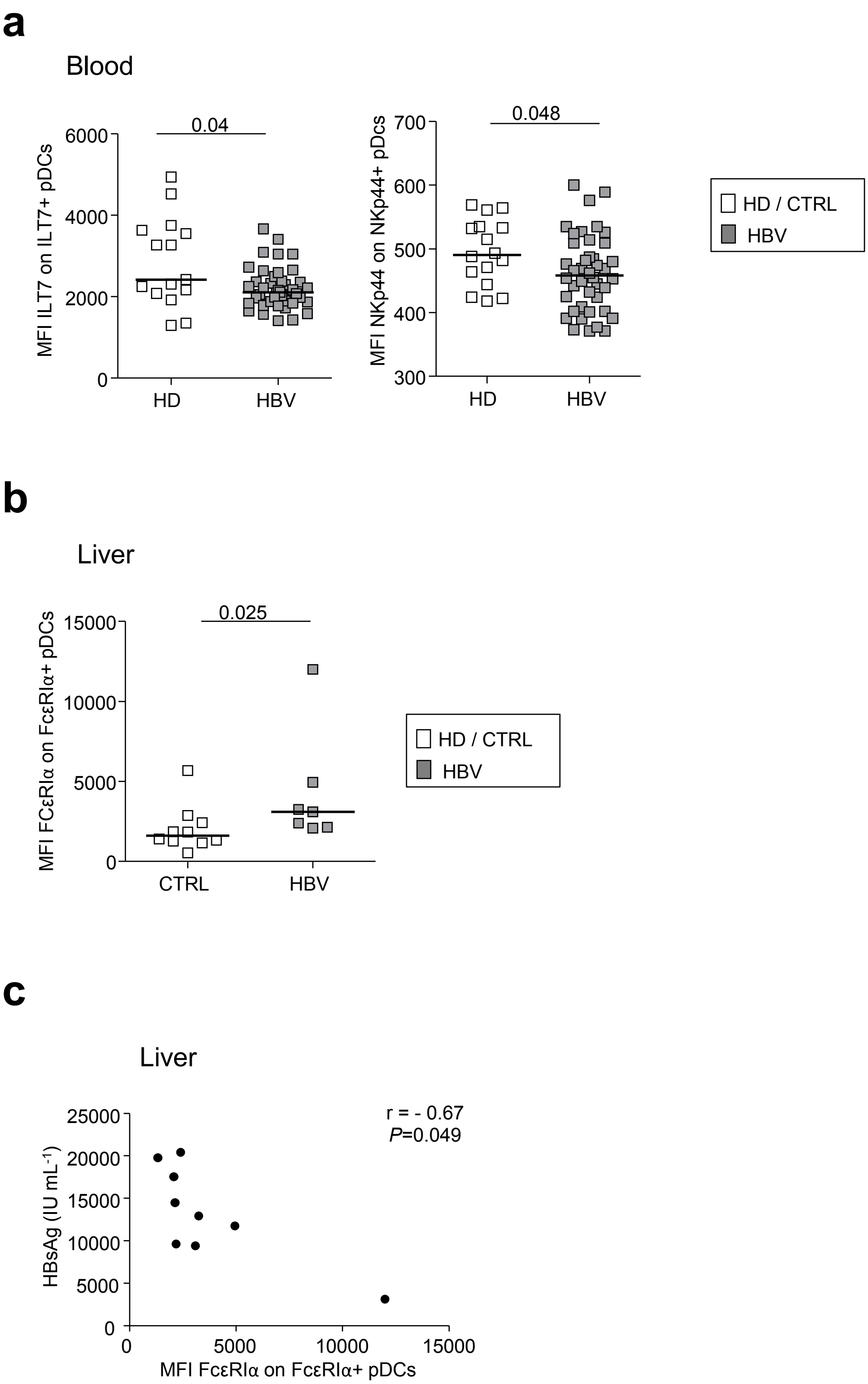

Supplementary Figure 4

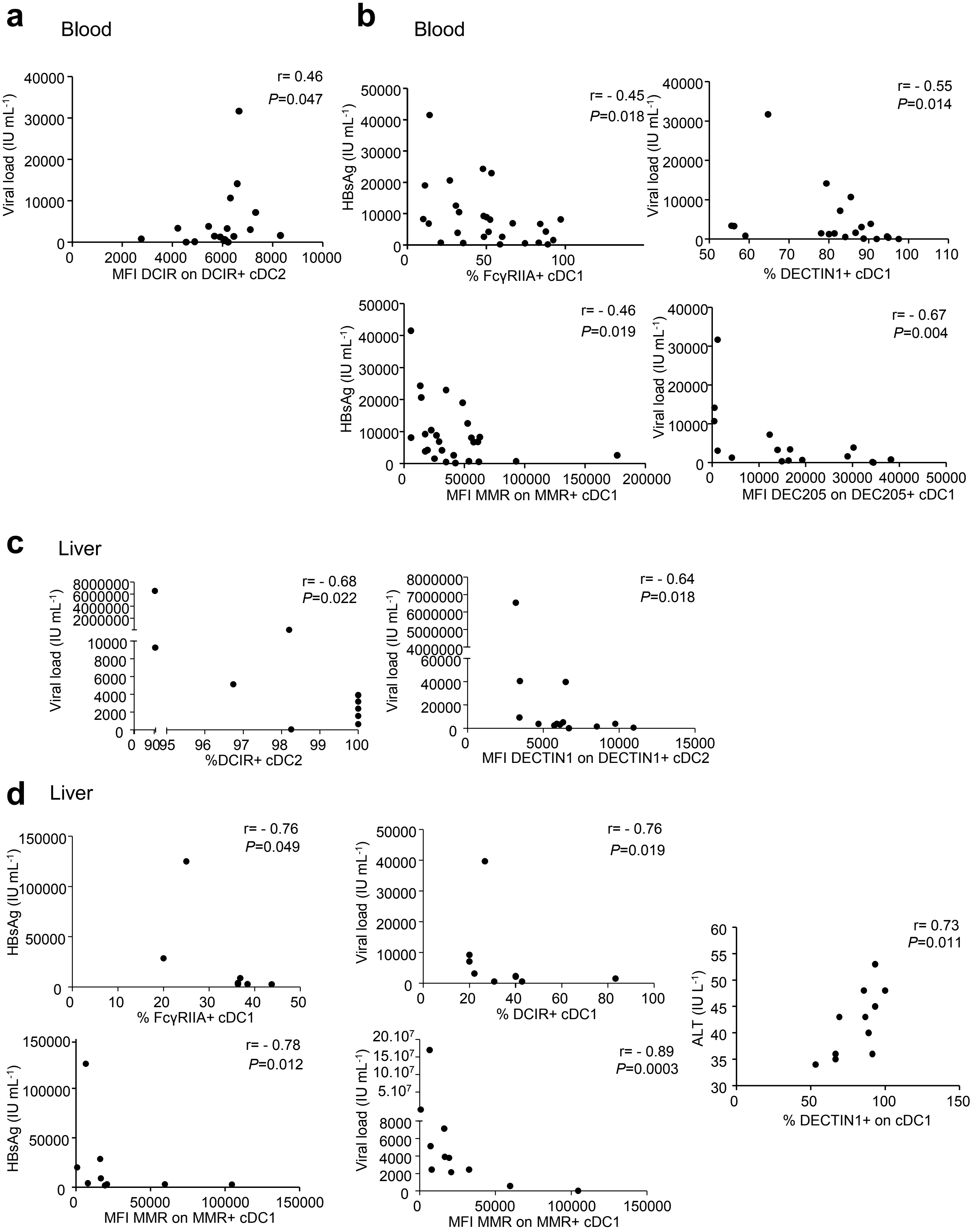

Supplementary Figure 5

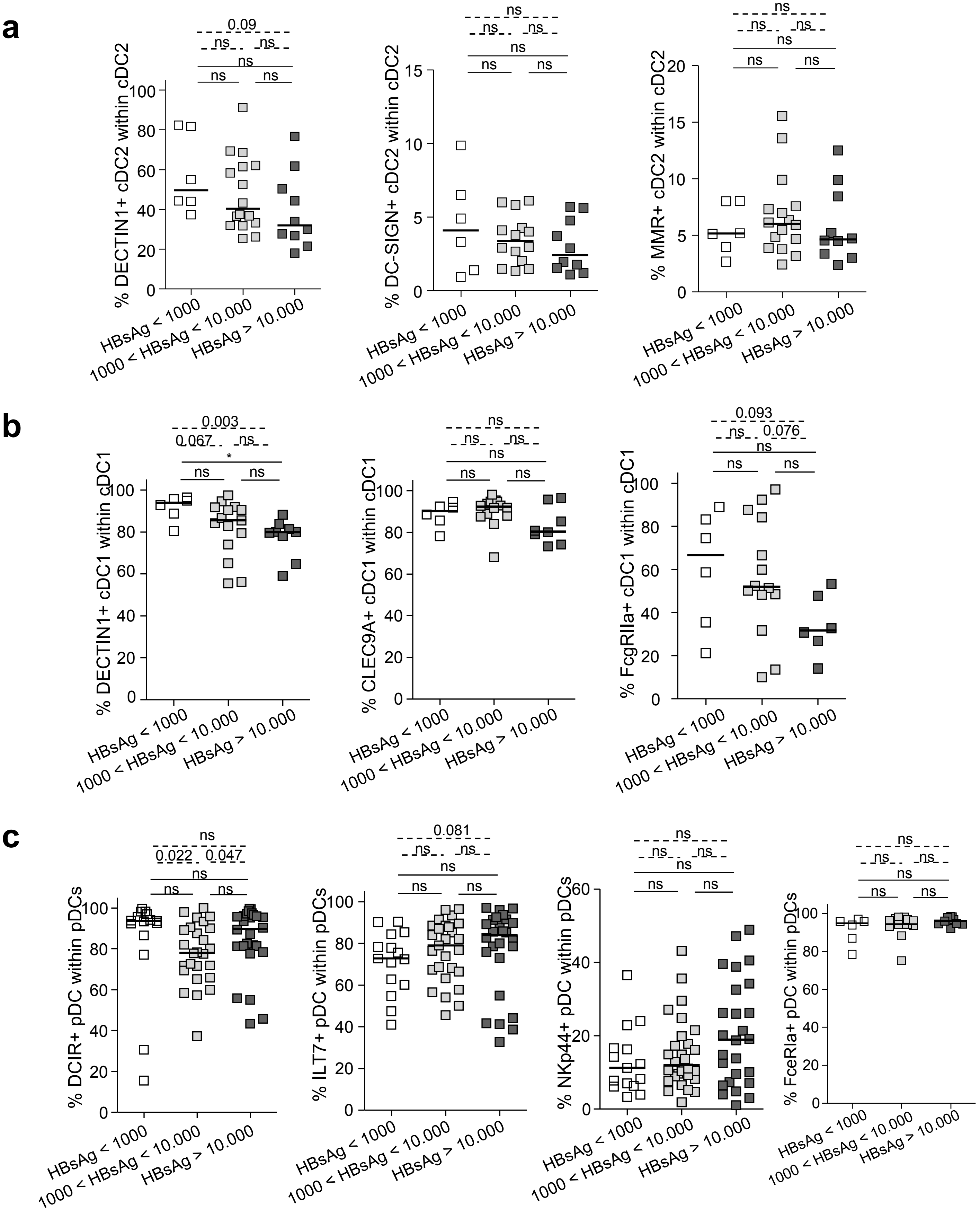

Supplementary Figure 6

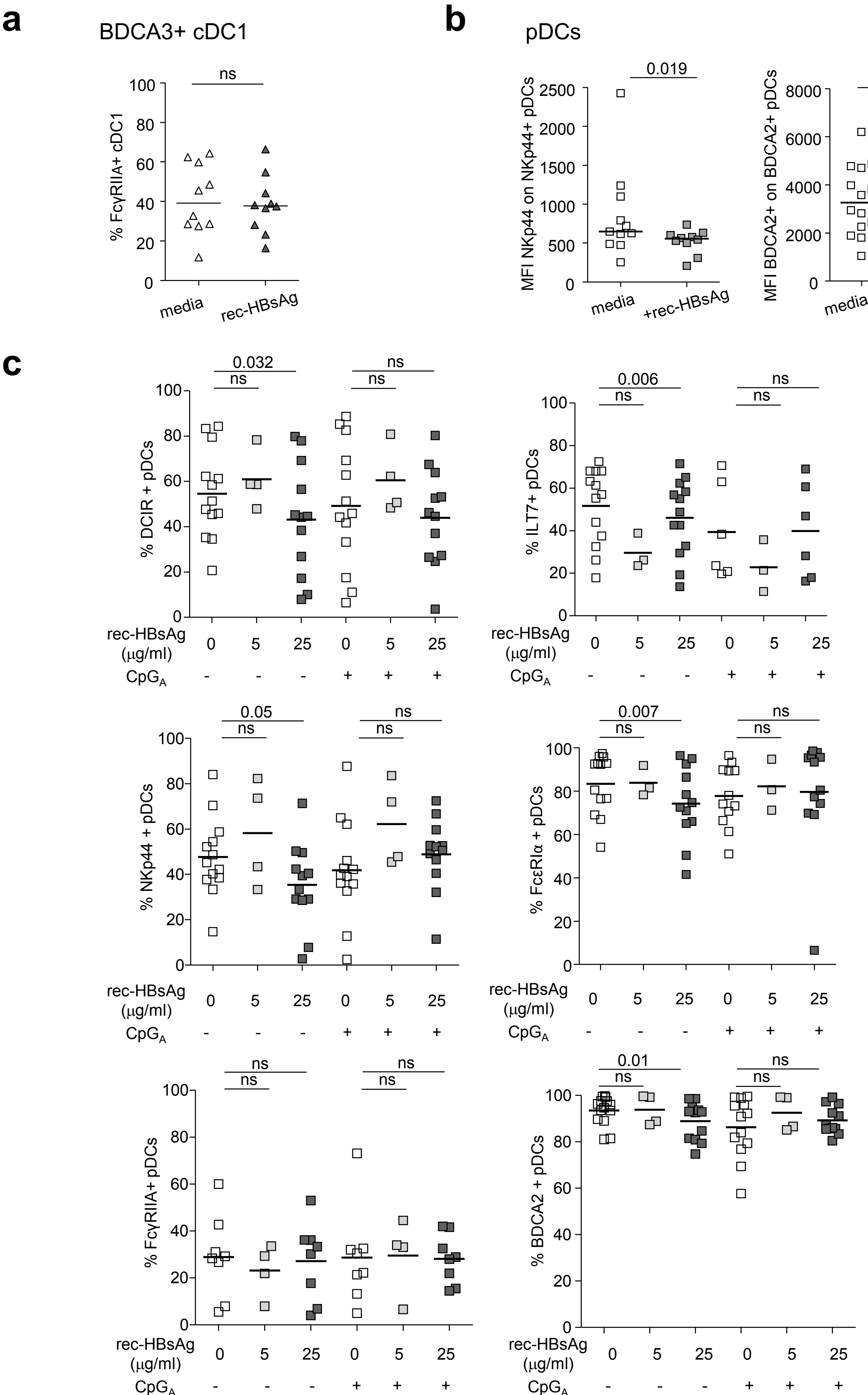

Supplementary Figure 7

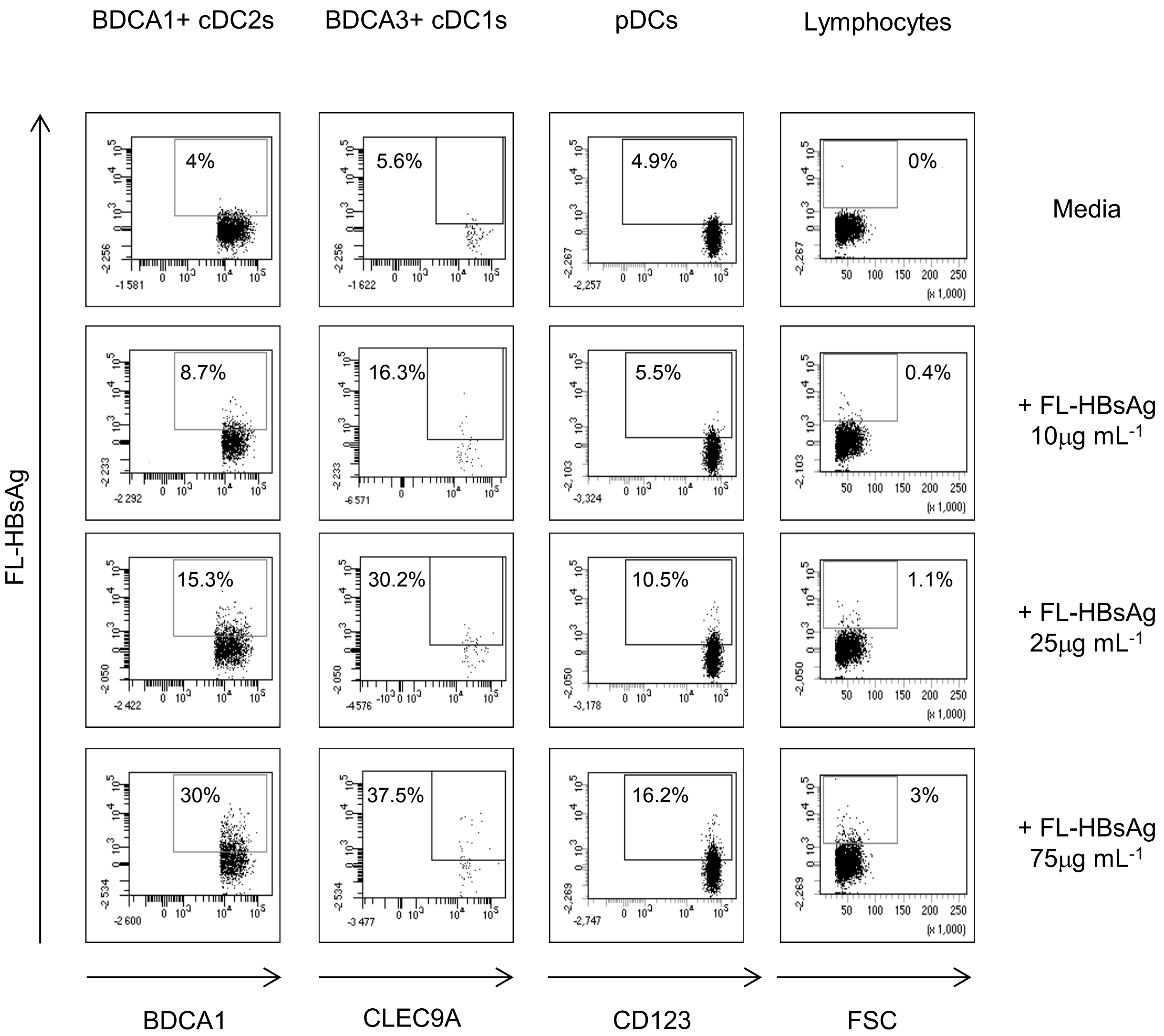

Supplementary Figure 8

a

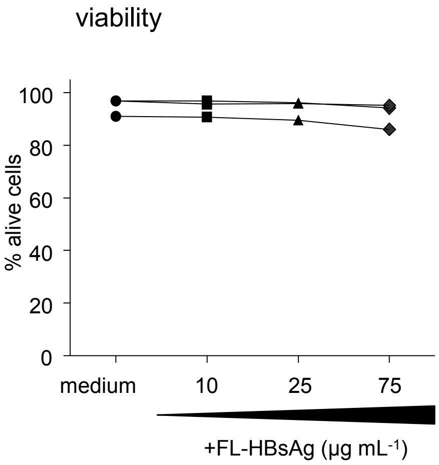

b

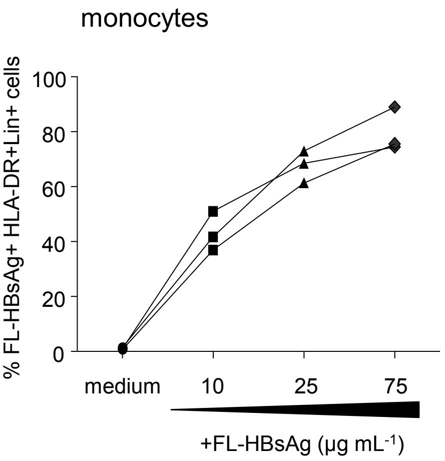

Supplementary Figure 9

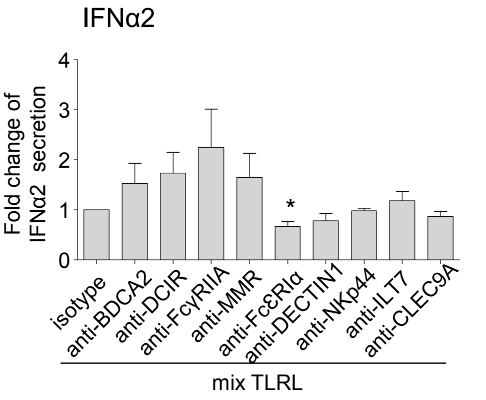

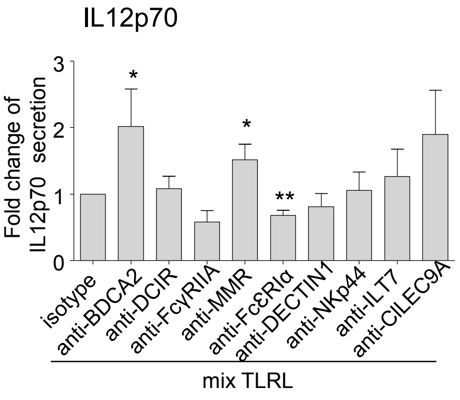

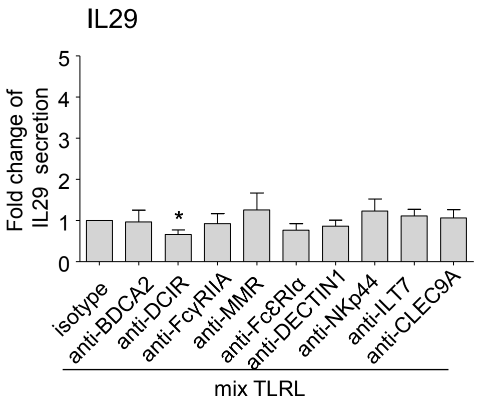

Supplement: Supplementary file 1 [file CTI2-9-e1208-s001.pdf]
